# Thermally Triggered Self-Reinforcing Double-Network Nanocomposite Hydrogels for Fracture Sealing Under Cyclic Steam Exposure

**DOI:** 10.3390/gels12090853

**Published:** 2026-09-19

**Authors:** Guangzhi Cui, Bin Li, Linpeng Zhang, Shuchen Yan, Sibo Wang, Jinjun Huang

**Affiliations:** 1 Exploration and Development Research Institute, PetroChina Liaohe Oilfield Company, Panjin 124010, China; 2 Oil Production Technology Research Institute, PetroChina Liaohe Oilfield Company, Panjin 124010, China; 3 State Key Laboratory of Oil and Gas Reservoir Geology and Exploitation, Southwest Petroleum University, Chengdu 610500, China

**Keywords:** covalent–physical double-network hydrogel, Laponite, thermal self-reinforcement, cyclic steam exposure, dynamic network reorganization, fracture sealing, heavy-oil thermal recovery

## Abstract

Hydrogels used for fracture sealing during heavy-oil thermal recovery are repeatedly exposed to high-temperature steam, dehydration, pressure disturbance, and cooling, conditions that often lead to progressive network deterioration and loss of sealing efficiency. To address this limitation, a thermally triggered self-reinforcing covalent–physical double-network nanocomposite hydrogel was developed by combining a sparse MBAA-crosslinked P(NaAMPS-co-NVP-co-AM) covalent scaffold with a reversible Laponite-mediated physical network. The material response was systematically evaluated through cyclic hydrothermal treatment at 180 °C, while fracture-sealing and resealing performance was examined separately under steam-exposure conditions. Artifact-controlled experiments were further introduced to distinguish genuine cycle-induced reinforcement from dehydration, continued post-curing, and conventional thermal aging. The results show that the NaAMPS/NVP/AM molar ratio of 40/20/40 with 1.00 wt% Laponite provided a suitable balance between precursor processability, hydrothermal stability, and mature network stiffness. After five thermal cycles at 180 °C, the storage modulus of the Lap-DN hydrogel increased from 74.4 to 89.1 kPa, while the compressive stress at 50% strain increased from 0.641 to 0.842 MPa, corresponding to reinforcement ratios of 1.198 and 1.314, respectively. Meanwhile, the mean equivalent pore diameter decreased from 6.2 to 4.8 μm, indicating progressive refinement of the load-bearing network. The hydrogel retained 76.3% of its initial water and 81.6% of its initial volume after five cycles in the reference brine and preserved a cycle-5 modulus retention of 101.3% even at 150,000 mg/L salinity. In fracture-sealing tests, Lap-DN sustained a breakthrough pressure of 3.47 MPa and reduced fracture conductivity by 92.4% in a 1.60 mm fracture. More importantly, the same sealing body maintained a breakthrough pressure of 4.55 MPa after five steam impact–recovery cycles in a 1.20 mm fracture, corresponding to 106.1% of the first-cycle value, while 95.0% of the pre-breakthrough pressure resistance was restored within 30 min of cooling. These results are consistent with a cycle-induced reorganization of the Laponite-mediated physical constraints within the permanent covalent scaffold, although the transient dissociation and reassociation of individual polymer–Laponite junctions were not directly observed. Within this evidence-based interpretation, cyclic thermal perturbation is associated with mechanical reinforcement and repeated fracture resealing rather than acting solely as a damaging factor. This work provides a materials-design strategy for hydrogel sealing systems operating under cyclic steam and high-salinity conditions.

## 1. Introduction

Heavy oil and bitumen represent important unconventional petroleum resources, but their high viscosity and poor mobility under reservoir conditions make efficient production considerably more difficult than that of conventional crude oil. Steam-based thermal recovery remains one of the most widely applied approaches because heating can substantially reduce crude-oil viscosity and improve its displacement and flow characteristics. Cyclic steam stimulation (CSS), steam flooding, and steam-assisted gravity drainage (SAGD) have therefore been extensively implemented in heavy-oil and oil-sands reservoirs. Nevertheless, their performance is strongly affected by reservoir heterogeneity, shale barriers, bottom-water invasion, gravity segregation, and uneven steam-chamber development, all of which may promote preferential steam migration and reduce the effective swept volume [1,2,3]. Field and laboratory investigations have further demonstrated that well configuration, reservoir-fluid properties, and steam-injection conditions jointly control thermal-recovery efficiency, while reservoir-scale simulations and field statistics consistently identify steam-channel development and poor conformance as major limitations to sustained recovery performance [4,5,6]. In fractured or strongly heterogeneous reservoirs, high-conductivity pathways can accelerate ineffective steam circulation and intensify heat loss, making selective control of fractures and preferential channels particularly important. Materials used under such conditions must not only withstand elevated temperature and salinity but also preserve hydration, mechanical integrity, and pressure-bearing capability during repeated heating, cooling, and hydraulic disturbance.

Polymer gels have been widely investigated for conformance control because their hydrated three-dimensional networks can deform, occupy irregular fractures, and substantially reduce preferential flow. Considerable progress has been made in organically crosslinked gels, preformed particle gels (PPGs), and related polymeric systems for high-temperature and high-salinity reservoirs. Improvements in monomer composition, crosslinking chemistry, particle architecture, and swelling regulation have enabled many gel systems to retain structural integrity and plugging capability under harsh reservoir conditions [7,8,9,10]. More recently, the design objective has gradually shifted from simple thermal resistance toward recoverability after damage. Self-healing and re-crosslinkable gels based on reversible ionic interactions, interpenetrating structures, or secondary crosslinking have demonstrated that network reconstruction can restore part of the mechanical or plugging performance after disruption [11,12,13]. Similar concepts have been extended to re-crosslinkable PPGs, self-healing polyampholyte particles, fractured-reservoir remediation gels, and environmentally compatible polymer systems, showing that reversible interactions can provide useful structural recovery under high salinity and temperature [14,15,16,17]. These advances demonstrate that dynamic network reconstruction is feasible in reservoir gels. However, most existing studies still treat high temperature primarily as a degradation factor to be tolerated or as a one-time trigger for additional crosslinking. Repeated steam heating–cooling, which is intrinsic to cyclic thermal-recovery operations, has rarely been considered as a distinct material-history variable. Under such conditions, dehydration, continued post-curing, ionic screening, reversible-junction rearrangement, and mechanical damage may occur simultaneously. Consequently, an increase in modulus after thermal exposure cannot automatically be interpreted as genuine self-reinforcement unless these competing effects are explicitly separated.

Nanoparticle-mediated physical networks provide a promising route for constructing hydrogels that combine permanent structural integrity with dynamic adaptability. Laponite is particularly attractive because its nanoscale disk-like platelets possess a large specific surface area and multiple interaction sites, enabling adsorption and physical association with polymer chains. Laponite-containing hydrogel systems have shown improved thixotropy, rheological recovery, mechanical strength, and plugging performance, while high-temperature and high-salinity polymer gel studies have demonstrated that combining permanent covalent structures with reversible secondary interactions can enhance long-term reservoir adaptability [18,19,20,21]. Related dual-crosslinked and re-crosslinkable acrylamide-based materials have further confirmed that dynamic secondary junctions can regulate swelling, improve mechanical integrity, and promote reconstruction after placement or thermal activation [22,23]. In parallel, recent studies on wellbore-sealing gels and degradable liquid plugs have highlighted that bulk mechanical properties alone are insufficient to describe sealing performance; interfacial adhesion, penetration, mechanical interlocking, and the preservation of pressure-bearing pathways also play critical roles in maintaining sealing integrity [24,25].

From a broader materials-science perspective, Laponite-based nanocomposite hydrogels provide a useful platform for exploring such dynamic reinforcement mechanisms. Polymer–Laponite interactions can act as reversible physical junctions that redistribute stress, dissipate mechanical energy, and increase toughness, while the permanent polymer network preserves overall connectivity. Experimental studies on Laponite-containing hydrogels have demonstrated substantial improvements in mechanical robustness, network uniformity, and environmental tolerance, and recent reviews emphasize that platelet dispersion, polymer adsorption, edge–surface interactions, and tactoid restructuring collectively determine the formation and evolution of Laponite-mediated physical networks [26,27,28,29]. More generally, physically crosslinked double-network hydrogels exploit a combination of persistent and reversible constraints to achieve toughness, damage tolerance, and recovery, providing an increasingly important design strategy for responsive soft materials [30]. These developments suggest that cyclic thermal exposure may do more than simply degrade a nanocomposite hydrogel. If reversible polymer–Laponite junctions are able to dissociate, redistribute, and reassociate within a permanent covalent scaffold, repeated heating–cooling could potentially reorganize the physical network into a mechanically more effective state. However, whether this process produces genuine cycle-specific self-reinforcement, whether it can be distinguished from dehydration or post-curing, and whether such reconstruction can be translated into repeated fracture sealing under steam exposure remain unresolved.

In this study, a thermally responsive covalent–physical double-network nanocomposite hydrogel was constructed using a sparse MBAA-crosslinked P(NaAMPS-co-NVP-co-AM) covalent scaffold and a Laponite-mediated reversible physical network. A covalent-network-only hydrogel (CN) and a nano-SiO_2_-containing nanocomposite reference formulation (Si-NC) were employed to provide two complementary reference states: CN represented the polymer network without an inorganic nanophase, whereas Si-NC provided a mass-matched but chemically and morphologically distinct nanoparticle reference. Because spherical nano-SiO_2_ and platelet-like Laponite differ in surface chemistry, geometry, accessible surface area, and potential polymer-interaction sites, Si-NC was not regarded as a fully equivalent nanoparticle control. Rather, it was used to determine whether incorporation of a non-layered inorganic nanophase at the same nominal loading could reproduce the characteristic response of Lap-DN. The covalent-network composition and Laponite loading were first optimized according to hydrothermal stability, gelation kinetics, precursor processability, and mature viscoelasticity. The polymer–nanoclay interactions and initial multiscale network architecture were then characterized using FTIR, XPS, XRD, TEM, and cryo-SEM. Time-matched, continuous-isothermal, and cyclic thermal histories were subsequently compared under standardized hydration conditions to isolate the contribution of cyclic thermal history. Rheological reinforcement, compressive behavior, energy dissipation, structural recovery, Laponite-order evolution, pore-network reconstruction, water retention, volume stability, and salinity tolerance were subsequently evaluated. Finally, variable-aperture fracture tests and repeated steam impact–recovery–resealing experiments using the same sealing body were conducted to connect network reconstruction with pressure-bearing and repeated sealing performance. The purpose of this work was therefore not simply to develop a hydrogel capable of surviving high temperature but to determine whether cyclic hydrothermal heating can stimulate physical-network reconstruction and self-reinforcement and whether the resulting reinforced state can subsequently translate into repeated fracture sealing under steam-exposure conditions relevant to heavy-oil thermal recovery.

## 2. Results and Discussion

### 2.1. Formulation, Gelation, and Baseline Properties of the Nanocomposite Hydrogel

#### 2.1.1. Chemical-Network Composition Screening Under Hydrothermal Exposure

**Figure 1:**
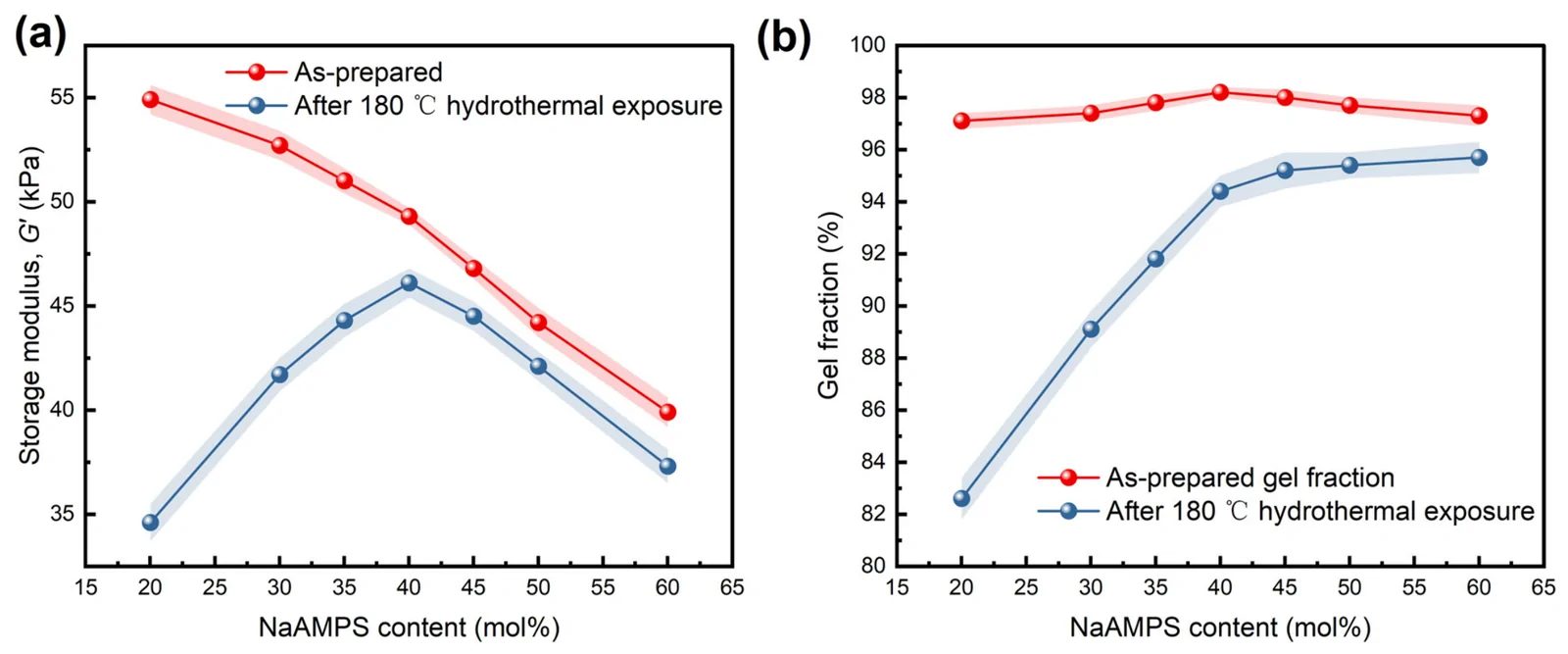
Hydrothermal screening of the permanent chemical-network composition. (**a**) Storage modulus (*G*′) of P(NaAMPS-co-NVP-co-AM) hydrogels with different NaAMPS/AM molar ratios before and after hydrothermal exposure at 180 °C. NVP was fixed at 20 mol%. (**b**) Gel fraction of the corresponding chemical networks before and after hydrothermal exposure. Error bars represent the standard deviations of three independently prepared samples.

The permanent network was optimized before introducing the Laponite-mediated physical network so that the thermal response of the base polymer could be evaluated independently of nanoparticle reinforcement. NVP was maintained at 20 mol%, whereas the NaAMPS/AM ratio was varied to adjust the balance between initial network stiffness and hydrothermal stability. Across the investigated composition range, the as-prepared storage modulus decreased from 54.9 to 39.9 kPa as the NaAMPS fraction increased from 20 to 60 mol% (Figure 1a). The behavior after hydrothermal exposure was distinctly different. Rather than decreasing monotonically, the retained modulus increased with NaAMPS content up to 40 mol%, where *G*′ reached 46.1 kPa after treatment at 180 °C, and then decreased again at higher NaAMPS fractions. The corresponding modulus retention at 40 mol% NaAMPS was 93.5%, compared with only 63.0% for the most AM-rich formulation.

The decline in initial modulus toward the NaAMPS-rich end means that a high retention ratio alone does not represent a mechanically preferable network. This is evident for the 45 and 50 mol% NaAMPS formulations: both retained slightly more than 95% of their initial *G*′, yet their post-treatment moduli were only 44.5 and 42.1 kPa, respectively, below the 46.1 kPa obtained for the 40/20/40 composition. Conversely, the AM-rich formulations possessed higher initial moduli but lost a much larger fraction of their stiffness during hydrothermal exposure. The optimum therefore did not coincide with either the highest initial modulus or the highest fractional retention. Instead, the 40/20/40 NaAMPS/NVP/AM composition occupied the point at which thermal retention and the absolute stiffness remaining after heating were simultaneously favorable.

The gel-fraction data provided a complementary measure of network integrity (Figure 1b). Before hydrothermal treatment, all formulations showed gel fractions between 97.1% and 98.2%, indicating that changes in the initial *G*′ were not caused by incomplete formation of the insoluble network. The difference became more pronounced after exposure to 180 °C. The gel fraction increased from 82.6% at 20 mol% NaAMPS to 94.4% at 40 mol%, after which the increase became marginal, reaching only 95.7% at 60 mol%. The plateau in gel fraction above approximately 40–45 mol% NaAMPS contrasts with the continued decrease in absolute *G*′ in the same composition range. Thus, preservation of the insoluble polymer fraction and preservation of network stiffness were not equivalent responses to hydrothermal exposure.

For the subsequent construction of the double-network hydrogel, the 40/20/40 composition was therefore selected as the permanent covalent network. This formulation maintained a high gel fraction after heating while providing the highest post-treatment storage modulus among the tested compositions. It also avoided shifting the formulation excessively toward NaAMPS at the expense of AM, which remains an important component of the polymer phase used later to establish polymer–Laponite physical associations. At this stage, however, the composition dependence is interpreted only at the level supported by rheological and gel-fraction measurements. Importantly, partial chemical modification of susceptible side groups during hydrothermal exposure would not necessarily result in dissolution of the hydrogel. The MBAA-crosslinked P(NaAMPS-co-NVP-co-AM) chains form a permanently connected covalent network, so local conversion of pendant functionalities, including possible partial hydrolysis of amide-containing segments, can alter hydration, electrostatic interactions, and local chain conformation without necessarily severing the polymer backbone or the covalent crosslinks. As long as backbone scission and crosslink cleavage remain limited, the modified chain segments remain tethered to the percolated network and the hydrogel retains macroscopic connectivity. The high post-treatment polymer gel fraction of the selected 40/20/40 composition (94.4%) is consistent with preservation of most of this insoluble network connectivity, even though local chemical changes cannot be excluded. The observed changes cannot be assigned specifically to hydrolysis, chain scission, or alteration of intermolecular interactions without additional structural evidence; these questions are addressed in the subsequent spectroscopic and morphological analyses.

#### 2.1.2. Formulation Window and Gelation Kinetics

**Figure 2:**
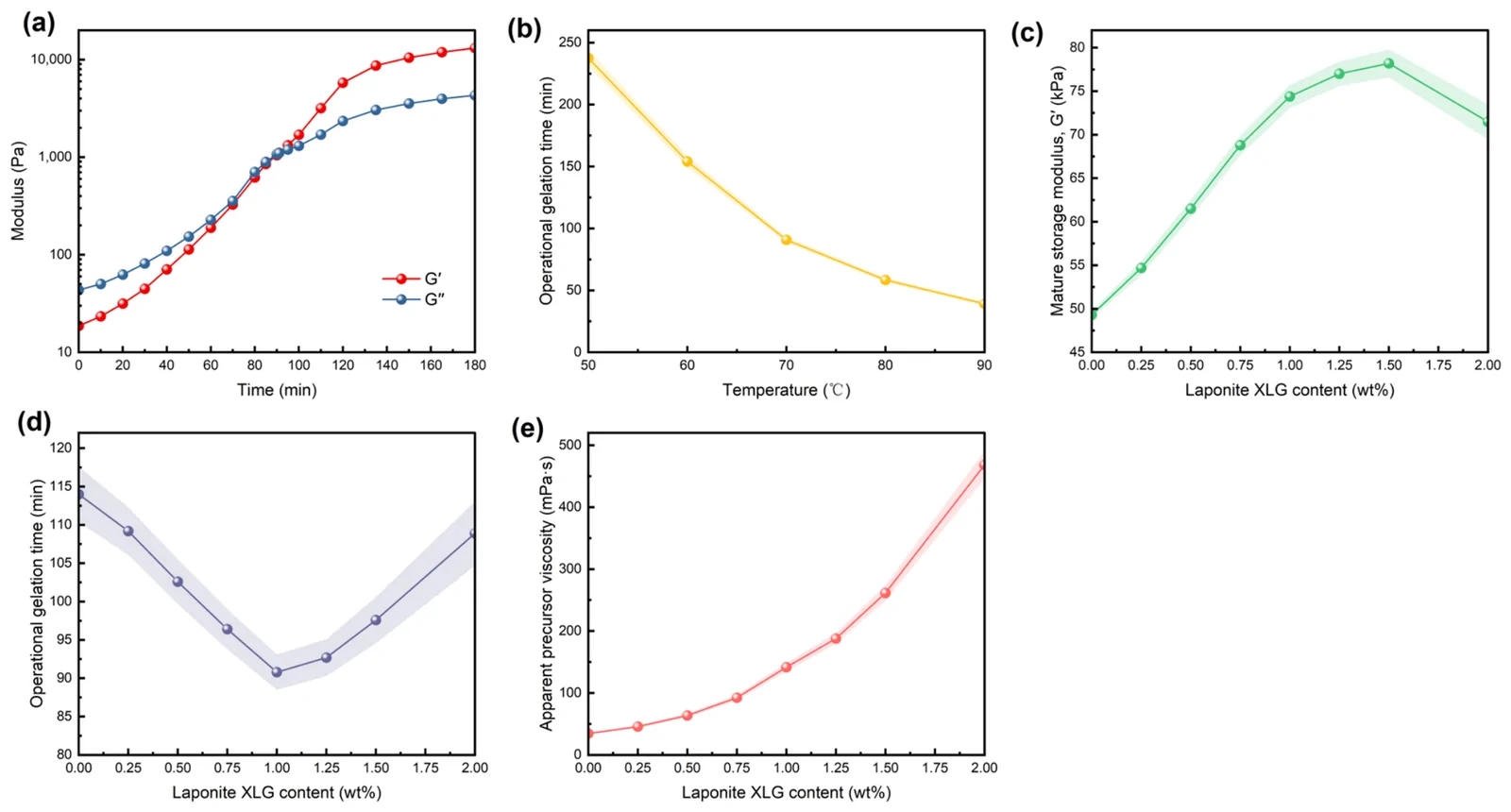
Formulation window and gelation kinetics of the Laponite-containing covalent–physical nanocomposite hydrogel. (**a**) Representative evolution of *G*′ and *G*′′ during gel formation of the formulation containing 1.00 wt% Laponite XLG at 70 °C. (**b**) Temperature dependence of the operational gelation time for the 1.00 wt% Laponite formulation. (**c**) Mature storage modulus as a function of Laponite XLG content. (**d**) Effect of Laponite XLG content on the operational gelation time at 70 °C. (**e**) Apparent precursor viscosity as a function of Laponite XLG content, measured at 25 °C and a shear rate of 10 s^−1^. Error bars in (**b**–**e**) represent standard deviations from three independently prepared samples.

After fixing the NaAMPS/NVP/AM molar ratio at 40/20/40, the Laponite content was varied to determine a composition range in which the secondary network could increase the mechanical contribution of the nanocomposite without producing an excessively viscous precursor. A representative rheological trace of the 1.00 wt% Laponite formulation is shown in Figure 2a. During the early stage of gelation at 70 °C, *G*′′ remained higher than *G*′, followed by a rapid increase in both moduli as network connectivity developed. The first persistent *G*′–*G*′′ crossover occurred at approximately 91 min. Beyond this point, *G*′ increased more rapidly than *G*′′ and reached approximately 1.32 × 10^4^ Pa at 180 min, confirming the transition to a predominantly elastic response. This value represents the modulus during the early curing period and should not be equated with the mature *G*′ measured after complete curing and equilibration.

The operational gelation time was strongly temperature dependent (Figure 2b). For the 1.00 wt% Laponite formulation, *t*_gel_ decreased from 237.6 min at 50 °C to 154.1 min at 60 °C and 90.8 min at 70 °C. Further increasing the temperature to 80 and 90 °C shortened *t*_g_ to 58.4 and 39.2 min, respectively. The nearly sixfold change over this temperature interval demonstrates that gel formation remains highly sensitive to the thermal history encountered before full network development. The *G*′–*G*′′ crossover is used here as an operational rheological criterion rather than a direct measure of vinyl conversion, because particle-mediated elasticity develops concurrently with covalent crosslinking in the Laponite-containing system.

The Laponite concentration altered the mature modulus over a much wider range than could be attributed to experimental scatter (Figure 2c). The nanoparticle-free chemical network exhibited a *G*′ of 49.3 kPa, consistent with the selected formulation identified in Figure 1. Addition of 0.25–1.00 wt% Laponite progressively increased *G*′ to 54.7, 61.5, 68.8, and 74.4 kPa. The increase became considerably smaller above 1.00 wt%: *G*′ reached 77.0 kPa at 1.25 wt% and 78.2 kPa at 1.50 wt%, corresponding to only a 5.1% increase relative to the 1.00 wt% formulation. At 2.00 wt%, the modulus decreased to 71.5 kPa. The loss of mechanical benefit at the highest loading indicates that increasing the inorganic fraction does not continuously improve network efficiency. Rheological data alone, however, cannot distinguish whether this deterioration originates from less uniform platelet dispersion, restricted polymer mobility, or both; this question is examined later using structural characterization.

The concentration dependence of *t*_g_ followed a similarly non-monotonic response (Figure 2d). Increasing the Laponite content from 0 to 1.00 wt% reduced the operational gelation time from 114.0 to 90.8 min. Above this concentration, the trend reversed: *t*_g_ increased to 92.7, 97.6, and 108.9 min at 1.25, 1.50, and 2.00 wt%, respectively. The initial decrease is consistent with an increasing contribution of particle-mediated elasticity to the evolving network, whereas the longer gelation times at higher loadings coincide with the concentration range in which the increase in mature *G*′ had already begun to level off. Because the rheological crossover reflects both covalent-network development and physical association, these changes should not be interpreted as direct evidence that Laponite accelerates or inhibits the underlying radical polymerization reaction.

A more restrictive limitation emerged from the precursor viscosity (Figure 2e). At 25 °C and a shear rate of 10 s^−1^, the apparent viscosity increased from 34.6 mPa·s without Laponite to 141.7 mPa·s at 1.00 wt%. Raising the Laponite content to 1.50 wt% increased the viscosity further to 261.6 mPa·s, despite providing only a modest additional increase in mature *G*′. At 2.00 wt%, the viscosity reached 468.3 mPa·s while the final modulus had already declined. The 1.00 wt% formulation therefore represented a practical composition boundary rather than the concentration giving the maximum modulus. It retained most of the mechanical benefit associated with Laponite addition, provided the shortest operational gelation time within the concentration series, and avoided the sharp viscosity increase observed at higher nanoparticle loadings.

Accordingly, 1.00 wt% Laponite XLG was selected for the subsequent thermal-cycling and structure–property experiments. This selection should not be interpreted as an optimization of stiffness alone. The formulation was chosen from the combined response of mature modulus, gelation behavior, and precursor rheology, leaving a network that remained sufficiently reinforced without moving into the high-solids regime where further Laponite addition produced diminishing mechanical returns.

#### 2.1.3. Baseline Physicochemical and Viscoelastic Properties

**Figure 3:**
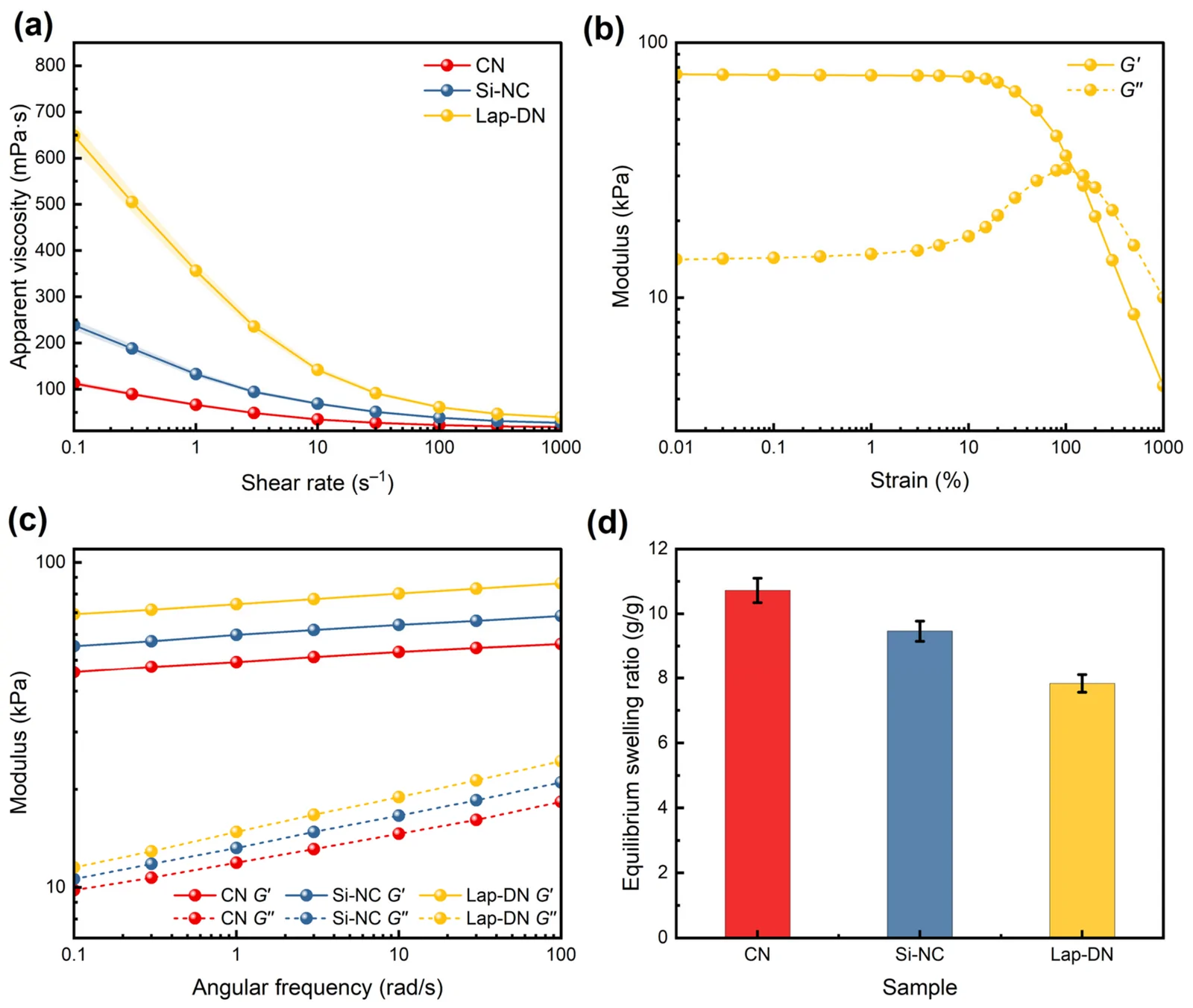
Baseline rheological and physicochemical properties of the optimized hydrogel systems. (**a**) Apparent viscosity of the CN, Si-NC, and Lap-DN precursors as a function of shear rate at 25 °C. (**b**) Amplitude sweep of the fully cured Lap-DN hydrogel at an angular frequency of 1 rad·s^−1^. (**c**) Frequency-dependent storage (*G*′) and loss (*G*′′) moduli of CN, Si-NC, and Lap-DN measured at 1% strain and 25 °C. (**d**) Equilibrium swelling ratios of the three hydrogel systems. Error bars in (**a**,**d**) represent standard deviations from three independently prepared samples; representative oscillatory rheological curves are shown in (**b**,**c**). A consistent formulation-specific color scheme is used throughout the figure: CN, red; Si-NC, blue; and Lap-DN, yellow-orange. In the oscillatory rheological plots, solid and dashed lines denote *G*′ and *G*″, respectively.

The optimized formulations already exhibited markedly different flow behavior before completion of the covalent network. Across the investigated shear-rate range, all three precursor systems showed decreasing apparent viscosity with increasing shear rate, but the magnitude of this response depended strongly on the inorganic phase (Figure 3a). At 0.1 s^−1^, the apparent viscosities of CN, Si-NC, and Lap-DN were 112.4, 238.6, and 648.5 mPa·s, respectively. At 10 s^−1^, these values decreased to 34.6, 68.9, and 141.7 mPa·s and further declined to 18.2, 27.2, and 38.9 mPa·s at 1000 s^−1^. Thus, although Laponite substantially increased the low-shear viscosity of the precursor, the difference between Lap-DN and the two control formulations became progressively smaller under high shear. The pronounced shear-thinning response is favorable for transporting a relatively structured precursor under imposed flow, while its higher viscosity at low shear provides a distinct rheological state once the applied shear is removed. At this stage, however, the flow curve is interpreted as a macroscopic rheological response rather than direct evidence for a continuous Laponite physical network.

The linear viscoelastic range of the fully cured Lap-DN was defined by an amplitude sweep before subsequent oscillatory measurements (Figure 3b). At low strain, G′ remained close to 74–75 kPa and was approximately five times greater than *G*′′, indicating an elasticity-dominated network under small deformation. Detectable departure from the low-strain *G*′ plateau began between approximately 15% and 20% strain. Beyond this range, *G*′ decreased progressively while *G*′′ increased, and the two moduli approached one another at strains of roughly 100–150%. The crossover occurred only after substantial disruption of the original network structure. A strain amplitude of 1% was therefore selected for subsequent oscillatory measurements.

The distinction among the three mature networks remained evident over the entire frequency window (Figure 3c). *G*′ exceeded *G*′′ from 0.1 to 100 rad·s^−1^ for CN, Si-NC, and Lap-DN, while both moduli increased only moderately with frequency. At 1 rad·s^−1^, *G*′ was 49.3 kPa for CN, 59.8 kPa for Si-NC, and 74.4 kPa for Lap-DN. The Laponite-containing network therefore exhibited a storage modulus approximately 50.9% higher than that of the covalent network and 24.4% higher than that of the SiO_2_-containing control under the same reference condition. The intermediate response of Si-NC is important: nanoparticle incorporation alone provides measurable reinforcement, but the additional increase obtained with Laponite cannot be reproduced by the spherical SiO_2_ control at the same inorganic loading. The relatively weak frequency dependence of *G*′ in Lap-DN further indicates that the mature material behaves as a persistent elastic network over the measurement timescale rather than as a transiently entangled polymer solution.

The increase in network stiffness was accompanied by a moderate reduction in equilibrium swelling (Figure 3d). The equilibrium swelling ratio decreased from 10.72 g/g for CN to 9.46 g/g for Si-NC and 7.83 g/g for Lap-DN. The corresponding equilibrium water contents were 91.5%, 90.4%, and 88.7%, respectively. The lower swelling ratio of Lap-DN is consistent with additional restrictions on network expansion after incorporation of the Laponite phase, although swelling data alone cannot distinguish physical crosslinking from other particle-induced constraints on chain mobility. Importantly, the reduction in swelling did not convert the material into a low-water-content polymer network; Lap-DN still contained nearly 89% water at equilibrium. This hydrated initial state provides an essential reference for the later steam-cycling experiments, where changes in modulus must be separated from those caused simply by water loss.

These baseline measurements define the state of the optimized hydrogel before thermal stimulation. Lap-DN combines a shear-thinning precursor, a broad small-deformation elastic regime, a higher mature *G*′ than either reference system, and a high equilibrium water content. The subsequent thermal-cycling analysis therefore evaluates changes relative to a network whose initial rheological and hydration states have already been independently established. Importantly, the contributions of the constituent network elements were resolved through a hierarchical control strategy rather than inferred from the final Lap-DN formulation alone. The covalent-network composition was first screened in the absence of inorganic nanoparticles; the Laponite loading was then varied at fixed polymer chemistry; and CN, Si-NC, and Lap-DN were compared at identical monomer and covalent-crosslinker contents. CN therefore isolates the contribution of the polymer network in the absence of an inorganic phase, whereas Si-NC provides a mass-matched non-layered nanoparticle reference. However, because nano-SiO_2_ and Laponite are not equivalent in surface chemistry, morphology, specific surface area, or density of polymer-accessible interaction sites, the Si-NC comparison is interpreted qualitatively rather than as a strictly matched nanoparticle control. The inability of Si-NC to reproduce the cycle-dependent response of Lap-DN therefore supports the importance of Laponite-specific structural features but does not by itself assign the response to any single interfacial interaction or exclude all possible nanoparticle-related effects.

### 2.2. Chemical Interactions and Initial Multiscale Network Architecture

#### 2.2.1. Spectroscopic Characterization of the Polymer Environment and Laponite Incorporation

**Figure 4:**
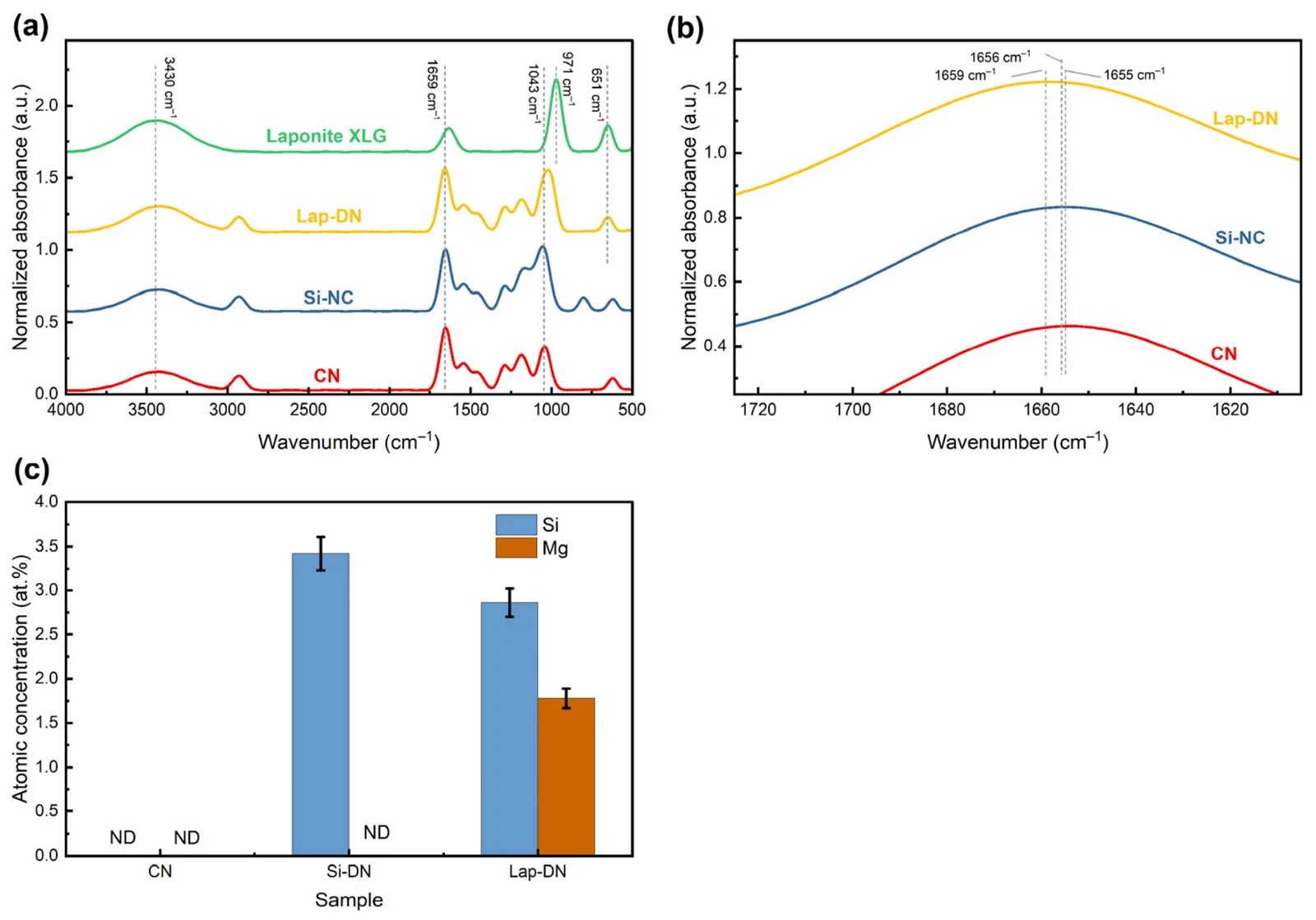
Spectroscopic characterization of the polymer environment and inorganic-phase incorporation in the nanocomposite hydrogels. (**a**) FTIR spectra of the covalent-network control (CN), nano-SiO_2_-containing reference formulation (Si-NC), Laponite-containing covalent–physical network (Lap-DN), and Laponite XLG. Spectra are vertically offset for clarity. (**b**) Enlarged amide-I region showing the subtle variation in the apparent carbonyl-band position among CN, Si-NC, and Lap-DN. (**c**) XPS-derived atomic concentrations of Si and Mg in CN, Si-NC, and Lap-DN. ND denotes concentrations below the instrumental detection limit. Error bars in (**c**) represent standard deviations from three independently prepared samples.

The chemical environment of the optimized hydrogel was examined after the formulation window had been fixed, with particular attention to whether Laponite behaved simply as an inert dispersed solid or interacted measurably with the polymer phase. The FTIR spectrum of CN retained the principal absorptions expected for the P(NaAMPS-co-NVP-co-AM) network, including the broad N–H/O–H envelope at approximately 3200–3500 cm^−1^, the amide-I/carbonyl band near 1655 cm^−1^, and the sulfonate-associated region between approximately 1000 and 1200 cm^−1^ (Figure 4a). Laponite XLG exhibited a strong silicate contribution near 970–1000 cm^−1^, together with lower-wavenumber features characteristic of the clay framework. Both polymer- and silicate-related absorptions were retained in Lap-DN. No distinct new absorption band was resolved after Laponite incorporation, which argues against interpreting the composite as being formed through a newly generated covalent linkage between the polymer and clay.

The most apparent difference was observed in the carbonyl region, where interference from the intense silicate and sulfonate bands was comparatively limited. The amide-I maximum of CN was located at approximately 1655 cm^−1^, while the corresponding band of Si-NC remained in a similar region at approximately 1656 cm^−1^ (Figure 4b). In Lap-DN, the amide-I maximum shifted to approximately 1659 cm^−1^. The approximately 4 cm^−1^ displacement relative to CN is small and is of the same order as the instrumental spectral resolution used in the present measurements. It is therefore treated as a subtle spectral perturbation rather than as independent evidence of a specific polymer–Laponite interaction. The near-overlap of the CN and Si-NC bands and the slightly displaced Lap-DN maximum are nevertheless compatible with a modest change in the average local environment of carbonyl-containing polymer segments after Laponite incorporation.

The amide-I envelope in this terpolymer contains overlapping contributions from AM and NVP carbonyl groups, and its position is also sensitive to local hydration and chain conformation. Accordingly, the small change in the amide-I maximum is interpreted only as being compatible with a perturbed local carbonyl environment in Lap-DN and is not used as proof of hydrogen bonding, specific adsorption, or any uniquely defined polymer–Laponite binding configuration. FTIR alone cannot resolve whether any interfacial association involves hydrogen bonding, electrostatic interactions, surface adsorption, changes in hydration, or a combination of these effects. Therefore, the FTIR result is used as supportive, but not definitive, evidence of a change in the local polymer environment after Laponite incorporation. FTIR was not used as a direct measure of hydrolysis or hydrothermal degradation. The spectra cannot quantify possible side-group hydrolysis or distinguish it from polymer-backbone scission. Therefore, preservation of macroscopic network integrity is inferred primarily from the high post-treatment gel fraction and retained mechanical response. Partial side-group modification, if present, would remain compatible with an insoluble hydrogel provided that the polymer backbone and a sufficient fraction of MBAA crosslinks remain intact.

XPS was used for a complementary purpose: to verify that the elemental signature of the inorganic phase was retained within the corresponding hydrogel rather than to infer bonding from small binding-energy shifts. Si and Mg were below the detection limit in CN, whereas Si-NC contained 3.42 at.% Si and no detectable Mg (Figure 4c). Lap-DN contained both Si and Mg, at 2.86 and 1.78 at.%, respectively. The simultaneous detection of these two elements is consistent with incorporation of the Laponite phase, while the absence of Mg from Si-NC provides a useful compositional distinction from the silica-containing control. The lower Si concentration in Lap-DN than in Si-NC is also reasonable because the two nanophases differ in elemental composition; the result does not require Lap-DN to exhibit the largest value for every inorganic-element signal.

Taken together, these measurements establish that Laponite is retained as an identifiable inorganic phase within Lap-DN. The FTIR data suggest only a subtle perturbation of the polymer carbonyl environment, whereas XPS independently verifies incorporation of the Laponite-derived inorganic phase. Neither technique, however, directly demonstrates a specific polymer–Laponite bonding configuration. Accordingly, evidence for the functional role of Laponite is evaluated collectively with the structural and control experiments rather than being assigned to the small FTIR peak displacement alone. The platelet dispersion state and the resulting multiscale network architecture are therefore examined separately in the following section by XRD and microscopic characterization.

In particular, the altered residual basal reflection observed by XRD, the mixed platelet/short-stack morphology observed by TEM, and the different rheological and pore-structural responses of Lap-DN relative to CN and the mass-matched Si-NC reference provide orthogonal evidence that Laponite modifies the network architecture, while still not constituting direct molecular-scale proof of a unique interfacial interaction.

#### 2.2.2. Laponite Dispersion and Initial Pore-Network Architecture

**Figure 5:**
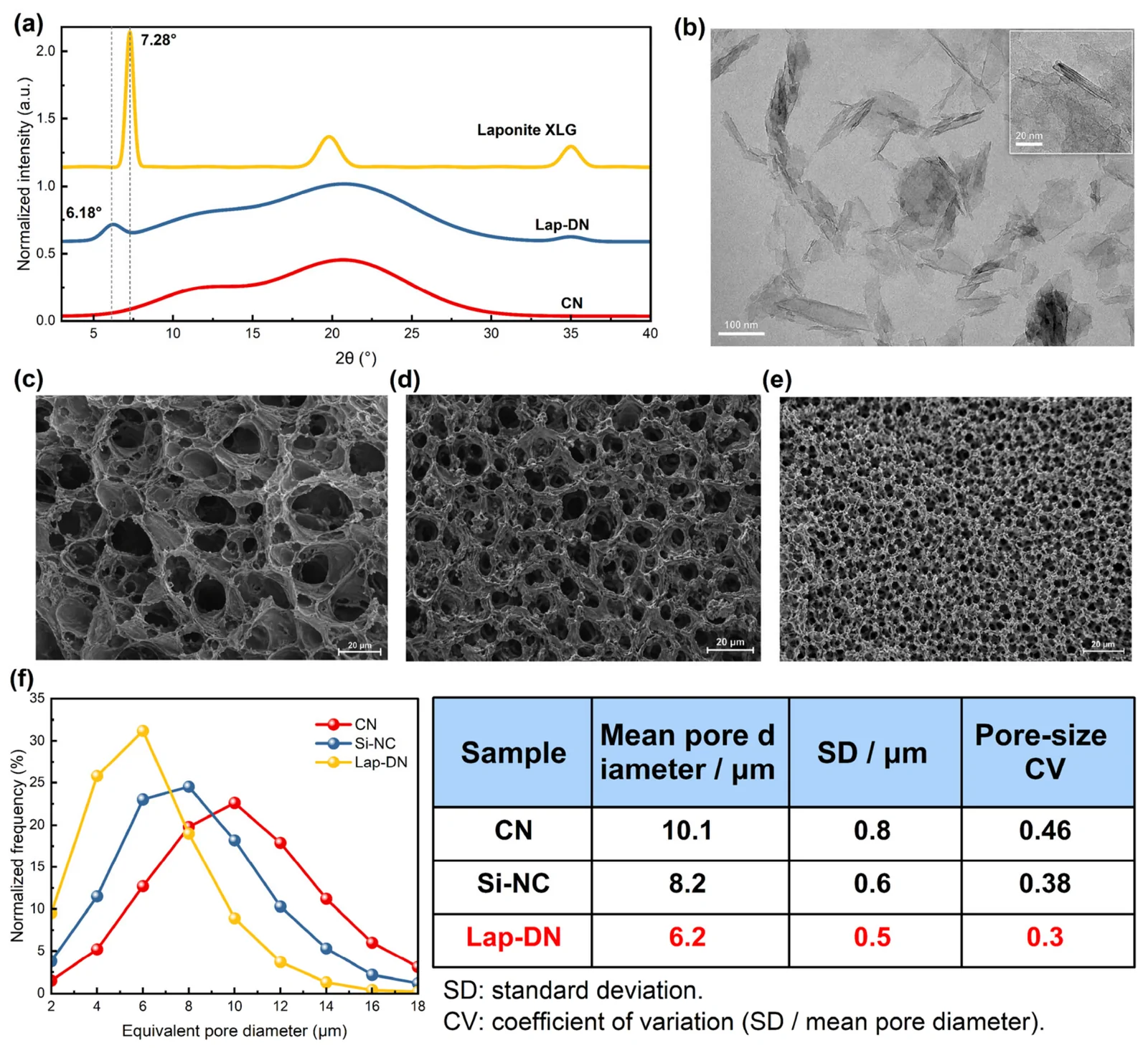
Laponite dispersion and initial pore-network architecture of the nanocomposite hydrogels. (**a**) XRD patterns of the covalent-network control (CN), Lap-DN, and Laponite XLG, highlighting the low-angle basal reflection of the layered nanosilicate. (**b**) Representative TEM image of the Laponite-containing precursor dispersion before polymerization, showing dispersed platelet-like domains, short stacks, and occasional tactoid-like structures. (**c**–**e**) Cryo-SEM images of CN, Si-NC, and Lap-DN, respectively, acquired under identical imaging conditions. (**f**) Equivalent pore-size distributions derived from cryo-SEM image analysis. Pore-size distributions are shown for visualization, whereas statistical comparisons are based on batch-level means from three independent hydrogel preparations.

The residual layered order of Laponite within the optimized hydrogel was examined by XRD as an ex situ comparative probe of platelet stacking in identically prepared dried specimens. Because the hydrogel samples were frozen and lyophilized before XRD analysis, the resulting low-angle diffraction features represent the residual ordered domains preserved after sample preparation and should not be interpreted as direct measurements of the native hydrated Laponite structure. Neat Laponite XLG showed a distinct (001) reflection at 2*θ* = 7.28°, corresponding to an interlayer spacing of 1.213 nm (Figure 5a). This value is consistent with reported Laponite XLG basal spacings of approximately 1.2 nm in the dry layered state. In contrast, CN displayed only broad amorphous features associated with the polymer network and no low-angle basal reflection.

After incorporation into the hydrogel, the sharp Laponite (001) reflection was no longer retained in its original form. Lap-DN exhibited only a weak and substantially broadened low-angle feature centered near 6.18°, corresponding to an apparent dried-state basal spacing of 1.429 nm. Its full width at half maximum increased from approximately 0.48° for neat Laponite to 1.16° in Lap-DN. The lower-angle position, reduced intensity, and pronounced broadening indicate that the residual coherently ordered Laponite domains in the lyophilized Lap-DN differ substantially from those in neat Laponite. However, because the reflection is weak and broad and because freeze-drying may alter clay hydration and stacking, these features cannot uniquely establish exfoliation or a specific platelet-rearrangement pathway in the hydrated hydrogel. The XRD result is therefore interpreted more conservatively as evidence of reduced or modified residual layer ordering in the dried composite. The platelet-dispersion state is assessed jointly with TEM rather than inferred from the basal reflection alone.

TEM observation of the Laponite-containing precursor provided complementary information on the platelet dispersion state before polymerization (Figure 5b). Thin platelet-like domains were distributed throughout the observed regions, together with short stacked structures and occasional tactoid-like domains. This mixed dispersion state is consistent with substantial disruption of the original Laponite stacking while retaining a limited population of residual stacked structures.

The platelet-level dispersion was accompanied by a measurable change in the micron-scale pore network. The nanoparticle-free CN showed the broadest pore-size distribution, with a batch-averaged equivalent pore diameter of approximately 10.1 μm (Figure 5c,f). Incorporation of nano-SiO_2_ reduced this value to 8.2 μm, whereas Lap-DN exhibited the smallest mean pore diameter, 6.2 μm. The reduction from CN to Lap-DN was therefore about 38.6%. More importantly, the pore-size coefficient of variation decreased from 0.46 for CN to 0.38 for Si-NC and 0.30 for Lap-DN, indicating that the Laponite-containing network was not simply denser but also more spatially uniform.

The pore-size distribution in Figure 5f illustrates the same trend without relying only on the mean value. CN was dominated by pores in the 8–14 μm range, while Si-NC shifted toward approximately 6–10 μm. Lap-DN showed its highest frequency at approximately 4–6 μm and contained relatively few pores larger than 12 μm. This narrowing is consistent with the lower equilibrium swelling ratio measured previously for Lap-DN and with its higher small-strain storage modulus. The three measurements therefore describe the same structural change from different scales: Laponite addition reduces unrestricted network expansion, increases the density of load-bearing constraints, and produces a finer initial pore architecture.

The intermediate behavior of Si-NC is also informative. Nano-SiO_2_ reduced the average pore size and increased the modulus relative to CN, showing that incorporation of an inorganic nanophase can itself modify the polymer-network architecture and mechanical response. Lap-DN, however, produced a larger reduction in pore size, a narrower distribution, and a greater increase in *G*′ at the same nominal nanoparticle loading. Because SiO_2_ and Laponite differ substantially in particle geometry, surface chemistry, accessible surface area, and polymer-interaction sites, the magnitude of this difference cannot be assigned uniquely to one material parameter. Nevertheless, the fact that the mass-matched Si-NC reference does not reproduce the Lap-DN response indicates that the behavior of Lap-DN cannot be explained by nanoparticle mass loading alone. Its stronger effect is therefore interpreted as arising from the combined consequences of the platelet morphology and interfacial characteristics of Laponite rather than from a generic filler effect. However, the present structural data still describe only the initial network architecture. Whether these platelet-mediated constraints can reorganize reversibly during thermal cycling is addressed later through artifact-controlled rheological, mechanical, and structural evolution measurements.

### 2.3. Artifact-Controlled Thermal Self-Reinforcement Under Cyclic Hydrothermal Treatment

#### 2.3.1. Discriminating Self-Reinforcement from Dehydration and Post-Curing

An increase in storage modulus after high-temperature hydrothermal treatment would not, by itself, constitute evidence of self-reinforcement. Hydrogel stiffening can arise simply from solvent loss, while incomplete curing can produce a similar response if residual reactive species continue to participate in network formation during high-temperature exposure. The thermal-history controls were therefore designed so that these contributions could be separated before the cycle-dependent response was interpreted mechanistically. The cyclic treatment consisted of five 30 min exposures at 180 °C, in the sealed high-pressure reactor under autogenous hydrothermal pressure, each followed by a standardized 30 min cooling/recovery period at 25 °C. No external gas pressure was imposed during the hydrothermal treatment; pressure developed from heating of the closed aqueous system and was not treated as an independently controlled experimental variable. The isothermal control received the same cumulative isothermal holding time of 150 min at 180 °C, whereas the time-matched control was maintained at 25 °C for the same total elapsed time required for the complete cyclic protocol, including the heating and cooling transitions.

**Figure 6:**
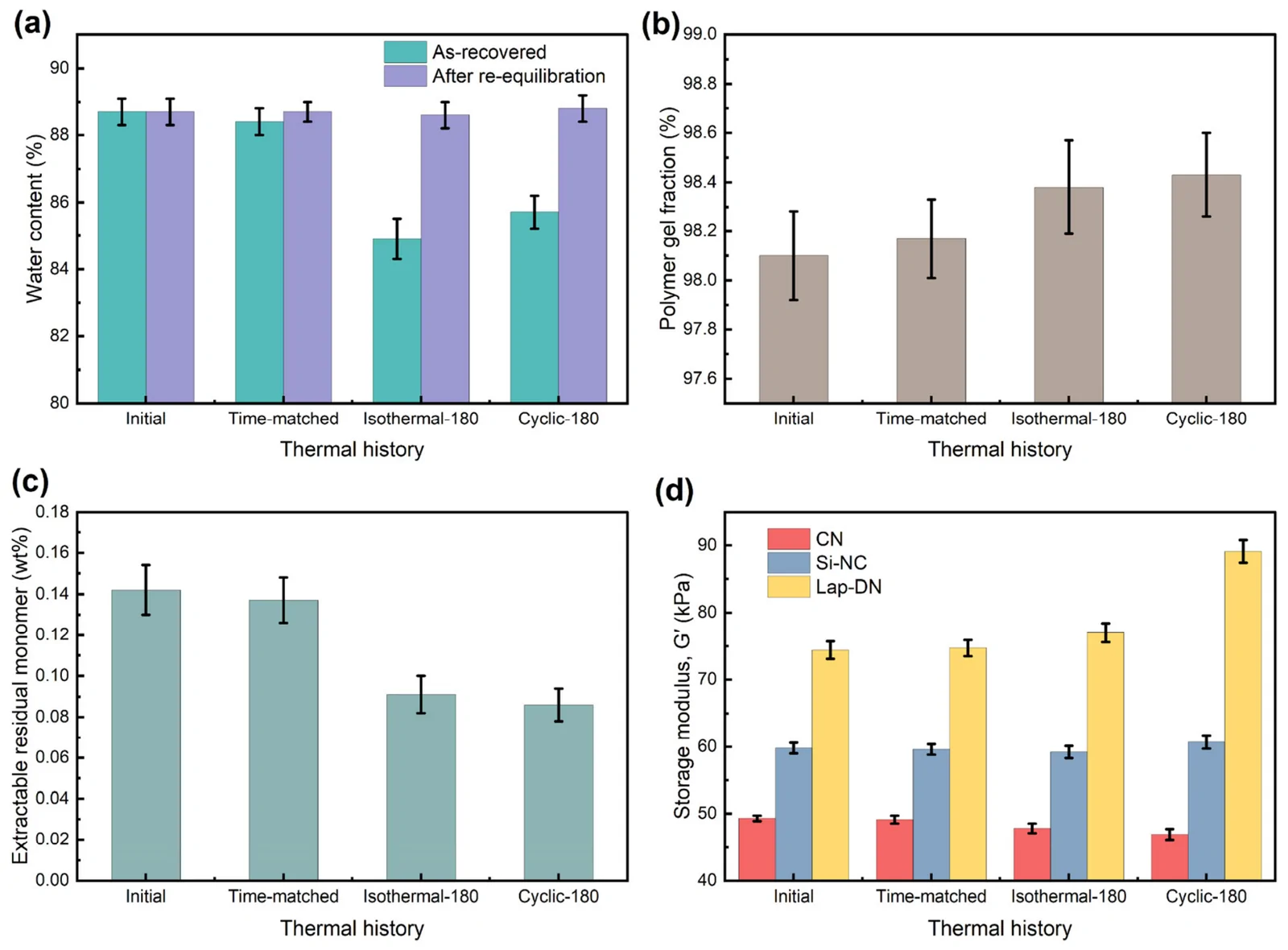
Control experiments used to distinguish cyclic-thermal reinforcement from dehydration and continued post-curing. (**a**) Water contents of Lap-DN immediately after the assigned thermal histories and after standardized re-equilibration in the same brine. (**b**) Polymer gel fraction of Lap-DN after different thermal histories, corrected for the inorganic Laponite mass. (**c**) Extractable residual-monomer content relative to the initial monomer charge. (**d**) Storage moduli of CN, Si-NC, and Lap-DN measured in the standardized post-treatment, re-equilibrated state at 25 °C, 1% strain, and 1 rad·s^−1^. The isothermal and cyclic groups received the same cumulative exposure time at 180 °C. Error bars represent standard deviations from three independently prepared samples.

Water loss was measurable immediately after high-temperature treatment but was not sufficient to account for the subsequent modulus increase (Figure 6a). The initial water content of Lap-DN was 88.7%. After treatment, the as-recovered values were 88.4% for the time-matched group, 84.9% after continuous exposure at 180 °C, and 85.7% after cyclic exposure. Re-equilibration restored the water contents to 88.6–88.8% in all groups. The modulus values used for comparison were measured only after this hydration normalization. The re-equilibration step was not assumed to preserve the instantaneous network configuration present immediately after high-temperature exposure. Because Lap-DN contains reversible physical constraints, immersion in fresh brine at 25 °C may permit additional hydration, chain relaxation, and redistribution or re-establishment of Laponite-mediated associations. Accordingly, the rheological measurements are interpreted as properties of the stabilized post-treatment, re-equilibrated state rather than as direct snapshots of the network at 180 °C or immediately after cooling. This point is particularly important because the isothermal sample actually showed slightly greater water loss than the cyclically treated sample before re-equilibration; a simple polymer-concentration effect would therefore predict at least comparable stiffening in the isothermal group, which was not observed.

Importantly, all thermal-history groups underwent the same 24 h re-equilibration protocol before rheological comparison. Therefore, although re-equilibration may contribute to the final physical-network configuration, it represents a common post-treatment condition and does not by itself account for the persistent difference between the isothermal-180 and cyclic-180 groups. The higher *G*′ retained by the cyclically treated samples after identical hydration normalization is thus interpreted as evidence of a thermal-history-dependent structural memory that survives standardized recovery, rather than as proof that the measured final structure was generated exclusively during the high-temperature holding stage.

The possibility of continued covalent-network formation was examined independently. The polymer gel fraction of Lap-DN changed only from 98.10% in the initial state to 98.38% after isothermal treatment and 98.43% after five thermal cycles (Figure 6b). These differences were small relative to the initial degree of network formation. The amount of extractable residual monomer was already low before thermal exposure, approximately 0.142 wt% of the initial monomer charge, and decreased to 0.091 and 0.086 wt% after isothermal and cyclic treatments, respectively (Figure 6c). Some additional consumption or removal of residual species during heating therefore cannot be excluded. However, the residual-monomer levels after the two 180 °C histories were nearly identical, as were their gel fractions, whereas their mechanical responses differed substantially. Continued curing alone cannot explain a cycle-specific increase of this magnitude.

That distinction becomes clear after the samples are compared at the same hydration state (Figure 6d). The *G*′ of Lap-DN was 74.4 kPa initially and remained essentially unchanged after the time-matched treatment at 74.7 kPa. Continuous exposure at 180 °C increased *G*′ only modestly to 77.0 kPa, corresponding to *G*′/*G*_0_′ = 1.035. In contrast, five heating–cooling cycles increased the re-equilibrated modulus to 89.1 kPa, giving *G*′/*G*_0_′ = 1.198. The same cyclic protocol did not produce a comparable response in the reference systems: CN decreased from 49.3 to 46.9 kPa, while Si-NC changed only from 59.8 to 60.7 kPa.

Because the cyclic and continuous treatments shared the same cumulative isothermal holding time of 150 min at 180 °C, and were subsequently subjected to the same 24 h hydration-normalization protocol, the difference between their responses cannot be attributed to high-temperature holding time alone, water loss, the common re-equilibration treatment alone, or the small amount of residual network conversion remaining after post-curing. At this stage, the result establishes that prior cyclic thermal history leaves a persistent reinforcement signature in the Laponite-containing network that remains detectable after standardized re-equilibration. It does not yet identify the microscopic origin of that response. Whether the increase arises from reversible rearrangement of polymer–Laponite junctions is addressed in the following section through cycle-resolved rheology and recovery measurements and later through direct comparison of the network structure before and after thermal cycling.

#### 2.3.2. Cycle-Dependent Rheological Reinforcement and Dynamic Recovery

**Figure 7:**
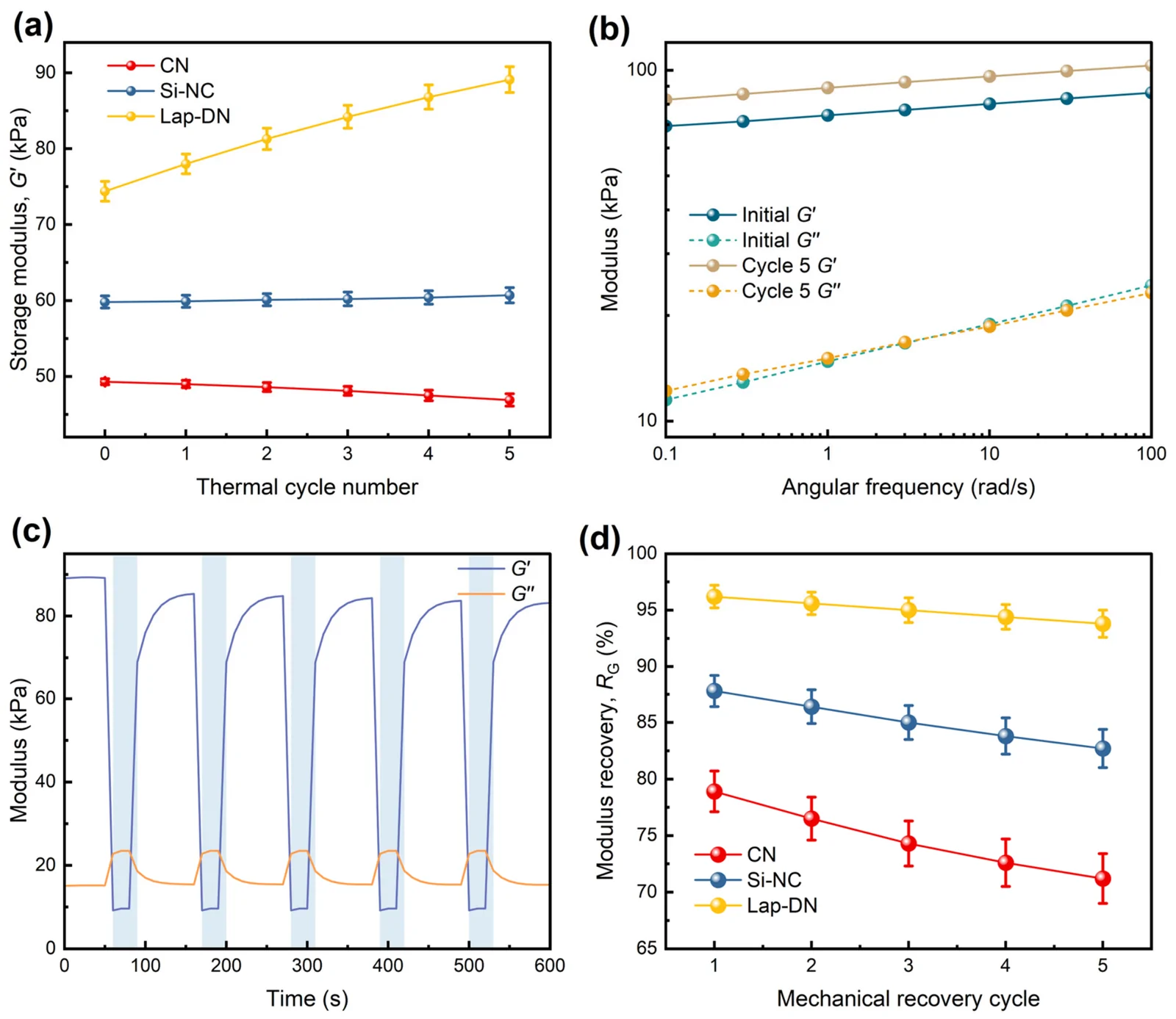
Cycle-dependent rheological reinforcement and dynamic recovery of the hydrogel networks. (**a**) Evolution of the storage modulus (*G*′) of CN, Si-NC, and Lap-DN with increasing thermal-cycle number. (**b**) Frequency-dependent *G*′ and *G*′′ of Lap-DN before thermal cycling and after five cycles. (**c**) Repeated step-strain response of Lap-DN after five thermal cycles under alternating strains of 1% and 300%; shaded regions denote the high-strain intervals. (**d**) Modulus-recovery ratio (*R*_G_) of CN, Si-NC, and Lap-DN over five repeated mechanical disruption–recovery cycles. Error bars in (**a**,**d**) represent standard deviations from three independently prepared samples.

The rheological response diverged progressively among the three networks as the number of thermal cycles increased. The storage modulus of CN decreased slightly from 49.3 kPa in the initial state to 46.9 kPa after five cycles, corresponding to *SRI*_G_ = 0.951 (Figure 7a). Si-NC remained comparatively stable, changing from 59.8 to 60.7 kPa over the same treatment. Lap-DN behaved differently. Its *G*′ increased successively from 74.4 kPa to 78.0, 81.3, 84.2, 86.8, and 89.1 kPa from cycles 0 to 5. The corresponding self-reinforcement index reached 1.198 after the fifth cycle. The increment became gradually smaller with successive cycles, from 3.6 kPa after the first cycle to 2.3 kPa between the fourth and fifth cycles, suggesting an approach toward a reinforced steady state rather than an unrestricted increase in network stiffness.

The reinforcement was not restricted to the single reference frequency used for cycle-by-cycle comparison. After five cycles, the *G*′ curve of Lap-DN shifted upward over the entire frequency range from 0.1 to 100 rad·s^−1^ (Figure 7b). At 1 rad·s^−1^, *G*′ increased from 74.4 to 89.1 kPa, whereas *G*′′ changed only slightly from 14.8 to 15.1 kPa. Accordingly, tan*δ* decreased from approximately 0.199 to 0.169. A similar separation between the storage and loss components was maintained at higher frequencies. The cycle-induced change is therefore expressed primarily as an increase in the elastic contribution of the mature network rather than as a proportional increase in both viscoelastic components.

Whether the reinforced network retained dynamic recoverability was examined by alternating the oscillatory strain between 1% and 300% after five thermal cycles (Figure 7c). At 1% strain, Lap-DN maintained *G*′ near 89 kPa with *G*′ > *G*′′. Increasing the strain to 300% caused *G*′ to fall to approximately 9–10 kPa while *G*′′ increased to approximately 23 kPa, reversing the modulus relationship and indicating disruption of the load-bearing network. Restoration of the strain to 1% led to a rapid recovery of *G*′, while *G*′′ returned toward its pre-damage level. The recovered *G*′ after the first high-strain event approached 85.7 kPa and remained approximately 83.6 kPa after the fifth disruption–recovery sequence. The reinforced state was therefore not associated with loss of recoverability under large deformation.

The distinction from the two control networks was clearer when recovery was quantified as

(1) $$R_{G}(n)\;=\;\frac{G_{\mathrm{rec},n}^{\prime }}{G_{\mathrm{pre}}^{\prime }}\;\times \;100\%$$

where *G*_pre_′ is the modulus immediately before the repeated high-strain sequence and *G*_rec,_*_n_*′ is the recovered modulus after the nth disruption event. Lap-DN recovered 96.2% of its pre-damage modulus after the first cycle and still retained 93.8% after the fifth mechanical recovery cycle (Figure 7d). The corresponding fifth-cycle values were 82.7% for Si-NC and 71.2% for CN. Thus, Laponite incorporation did not merely increase the absolute modulus; it also substantially reduced the irreversible loss accumulated during repeated large-strain disruption.

The combined cycle-resolved and step-strain results establish a rheological feature that is central to the present material design: repeated thermal cycling increases the elastic contribution of Lap-DN without eliminating its capacity for rapid structural recovery after large deformation. Given the artifact controls established in Section 2.3.1, this response is consistent with progressive reorganization of the Laponite-mediated physical junctions within the permanent covalent scaffold. The rheological measurements alone do not resolve the precise nanoscale rearrangement pathway, however, and the structural origin of this reinforced state is examined later by comparing the network architecture before and after thermal cycling.

### 2.4. Mechanical Self-Reinforcement, Energy Dissipation, and Recovery

#### 2.4.1. Cycle-Dependent Compressive Reinforcement

**Figure 8:**
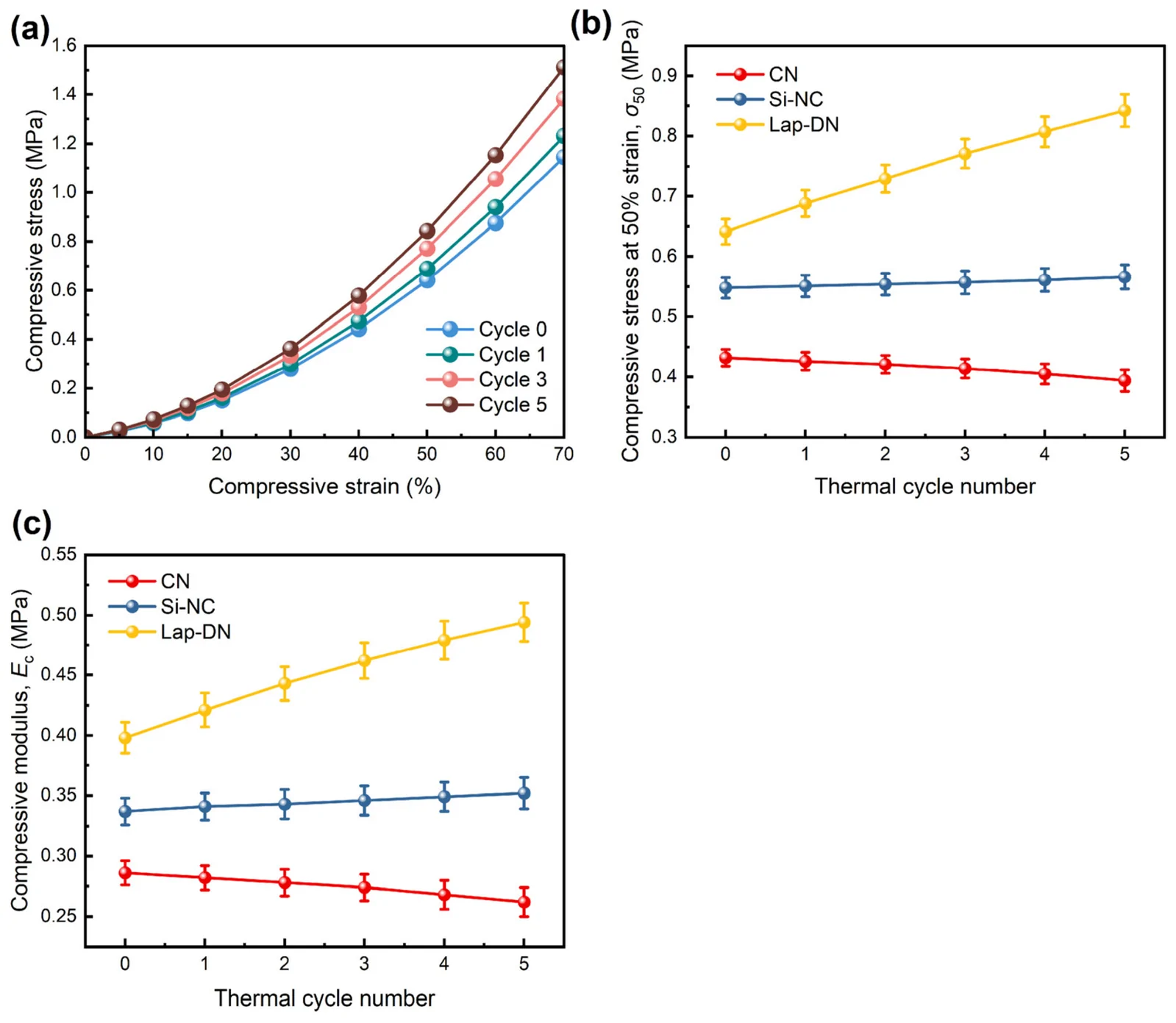
Cycle-dependent compressive reinforcement of the hydrogel networks. (**a**) Representative compressive stress–strain curves of Lap-DN before thermal treatment and after 1, 3, and 5 thermal cycles. (**b**) Evolution of the compressive stress at 50% strain (*σ*_50_) for CN, Si-NC, and Lap-DN with increasing thermal-cycle number. (**c**) Cycle-dependent compressive modulus (*E*_c_) obtained from the 5–15% strain region. Error bars in (**b**,**c**) represent standard deviations from three independently prepared specimens.

The cycle-dependent increase in *G*′ was accompanied by a clear change in the compressive response of Lap-DN. All specimens showed the nonlinear stress increase characteristic of a highly hydrated network under progressive compression, but the stress level at a given strain shifted upward after repeated thermal cycling (Figure 8a). At 50% strain, the compressive stress of Lap-DN increased from 0.641 MPa in the initial state to 0.688 MPa after the first cycle, 0.771 MPa after the third cycle, and 0.842 MPa after the fifth cycle. The difference became more pronounced at larger deformation: at 70% strain, the corresponding stresses increased from approximately 1.15 to 1.51 MPa. Thus, the thermally reinforced state was not restricted to the small-deformation regime probed by oscillatory rheology.

Comparison with the two reference systems further separated the Lap-DN response from ordinary thermal aging or nanoparticle reinforcement (Figure 8b). The *σ*_50_ of CN gradually decreased from 0.432 to 0.394 MPa over five cycles, indicating a modest loss of load-bearing capacity. Si-NC remained comparatively stable, increasing only from 0.548 to 0.566 MPa. Lap-DN, by contrast, showed a progressive increase from 0.641 to 0.842 MPa. Defining the compressive self-reinforcement index as

(2) $$SRI_{\sigma }(n)=\frac{\sigma_{50,n}}{\sigma_{50,0}}$$

the fifth-cycle value for Lap-DN reached 1.314, corresponding to a 31.4% increase relative to its initial state. This increase is larger than the approximately 19.8% rise in *G*′ observed after five thermal cycles, indicating that the reinforced network provides an even greater benefit once deformation extends beyond the linear viscoelastic range.

The small-strain compressive modulus showed the same directional response (Figure 8c). *E*_c_, obtained from the linear portion between 5% and 15% compressive strain, increased from 0.398 MPa initially to 0.494 MPa after five cycles for Lap-DN, corresponding to an increase of 24.1%. Over the same treatment, *E*_c_ decreased from 0.286 to 0.262 MPa for CN, whereas Si-NC changed only from 0.337 to 0.352 MPa. The simultaneous increase in both *E*_c_ and *σ*_50_ indicates that thermal cycling affects both the initial resistance to deformation and the load-bearing capacity developed at finite strain.

The mechanical response is therefore consistent with the rheological reinforcement established in Section 2.3, but the two measurements probe different deformation regimes. Oscillatory *G*′ reflects the integrity of the network under small, non-destructive deformation, whereas *σ*_50_ reflects its ability to sustain substantial structural compression. Their concurrent increase in Lap-DN, together with the absence of a comparable response in CN and Si-NC, supports the interpretation that repeated thermal cycling produces a mechanically more effective network rather than merely increasing the apparent small-strain stiffness. The manner in which this reinforced network dissipates energy and recovers after unloading is examined in the following section.

#### 2.4.2. Loading–Unloading Hysteresis and Structural Recovery

**Figure 9:**
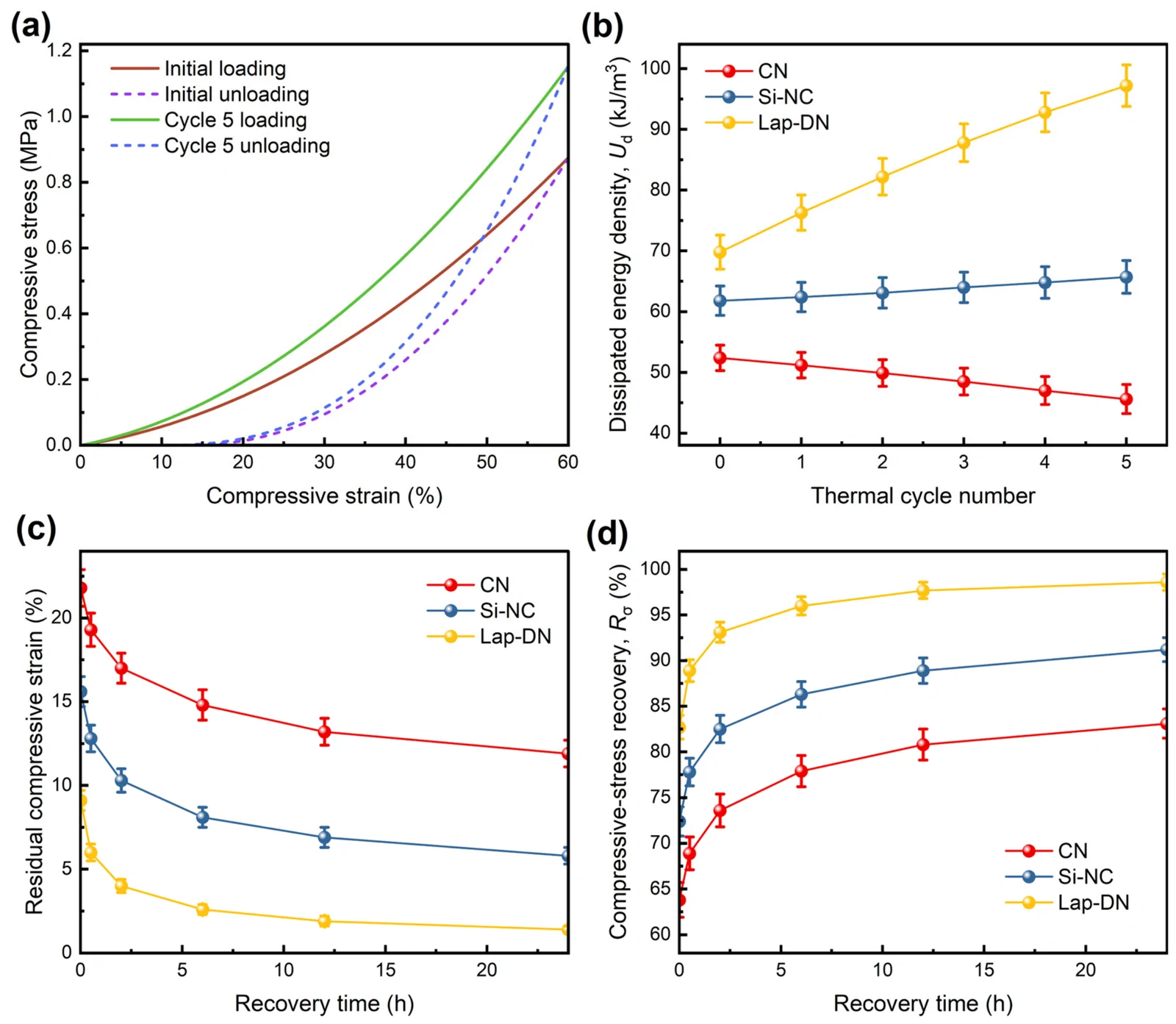
Loading–unloading hysteresis, energy dissipation, and structural recovery of the hydrogel networks. (**a**) Representative compression loading–unloading curves of Lap-DN before thermal cycling and after five thermal cycles at a maximum compressive strain of 60%. (**b**) Evolution of the dissipated energy density (*U*_d_) of CN, Si-NC, and Lap-DN with increasing thermal-cycle number. (**c**) Recovery of residual compressive strain after a single 60% compression applied after the fifth thermal cycle. (**d**) Recovery of the compressive stress at 50% strain, expressed as *R*_σ_, during resting recovery in the same aqueous medium at 25 °C. Error bars in (**b**–**d**) represent standard deviations from three independently prepared samples.

The increase in compressive strength after thermal cycling was accompanied by a marked change in the loading–unloading response of Lap-DN. Under compression to 60% strain, the initial hydrogel reached approximately 0.875 MPa, whereas the specimen after five thermal cycles reached 1.153 MPa (Figure 9a), consistent with the monotonic compression curves reported in Figure 8a. Both states exhibited pronounced hysteresis during unloading, indicating that part of the mechanical work was dissipated rather than stored elastically. The loop became larger after thermal cycling even though the immediate residual strain decreased from approximately 12.8% to 9.1%. Thus, the reinforced state did not simply behave as a stiffer network with greater permanent deformation; it accommodated a larger mechanical load while retaining a greater capacity to recover its macroscopic shape after unloading.

The dissipated energy density was quantified from the area enclosed by the compression loop,

(3) $$U_{d}\;=\;\int_{\mathrm{loading}}\sigma d\varepsilon \;-\;\int_{\mathrm{unloading}}\sigma d\varepsilon$$

For Lap-DN, *U*_d_ increased from 69.8 kJ/m^3^ before thermal cycling to 76.3, 82.2, 87.8, 92.8, and 97.2 kJ/m^3^ after successive cycles (Figure 9b). This represents an increase of approximately 39.3% after the fifth cycle. The increase cannot be attributed solely to the higher loading stress: the fraction of loading work dissipated within the loop, *U*_d_/*U*_load_, increased from approximately 35.1% to 37.3%. CN showed the opposite trend, with *U*_d_ decreasing from 52.4 to 45.6 kJ/m^3^, whereas Si-NC changed only slightly from 61.8 to 65.7 kJ/m^3^. The thermal-cycle response of Lap-DN therefore combines increased load-bearing capacity with enhanced capacity for mechanical energy dissipation.

Recovery continued after unloading rather than being completed instantaneously. Following a single 60% compression applied after the fifth thermal cycle, the immediate residual strains of CN, Si-NC, and Lap-DN were 21.8%, 15.6%, and 9.1%, respectively (Figure 9c). During resting recovery in the same aqueous medium at 25 °C, all three values decreased but at distinctly different rates. After 6 h, the residual strain of Lap-DN had fallen to 2.6%, compared with 8.1% for Si-NC and 14.8% for CN. After 24 h, Lap-DN retained only 1.4% residual strain, whereas Si-NC and CN remained at 5.8% and 11.9%, respectively. The smaller initial residual deformation and faster subsequent recovery indicate that the thermally reinforced Lap-DN network retains substantial elastic restoring capacity despite the larger hysteretic energy loss observed during compression.

Recovery of macroscopic shape was accompanied by restoration of load-bearing capacity. The compressive-strength recovery ratio was defined as

(4) $$R_{\sigma }(t)\;=\;\frac{\sigma_{50,\mathrm{rec}}(t)}{\sigma_{50,\mathrm{pre}}}\;\times \;100\%$$

where *σ*_50,pre_ is the compressive stress at 50% strain measured before the damaging compression, and *σ*_50,rec_(*t*) is the corresponding stress at 50% strain measured after recovery for time t. Immediately after unloading, Lap-DN retained 82.7% of its pre-compression *σ*_50_, compared with 72.4% for Si-NC and 63.8% for CN (Figure 9d). The recovery of Lap-DN reached 93.1% after 2 h and 96.0% after 6 h, approaching 98.6% after 24 h. Si-NC recovered to 91.2% over the same period, while CN remained at 83.1%.

The simultaneous increase in hysteretic dissipation and post-deformation recovery is consistent with the intended covalent–physical network architecture. A purely brittle strengthening mechanism would generally increase resistance to compression at the expense of recoverability, whereas Lap-DN retained both properties after repeated thermal treatment. The present mechanical data are consistent with a permanent covalent scaffold that preserves macroscopic connectivity while a population of reversible Laponite-mediated physical constraints participates in deformation, energy dissipation, and subsequent structural recovery. The exact interfacial rearrangement responsible for this behavior cannot be resolved from the mechanical measurements alone and is therefore examined further through the structural evolution of the network during thermal cycling.

### 2.5. Cycle-Induced Structural Evolution and Hydrothermal Property Retention

#### 2.5.1. Multiscale Structural Evolution During Thermal Cycling

**Figure 10:**
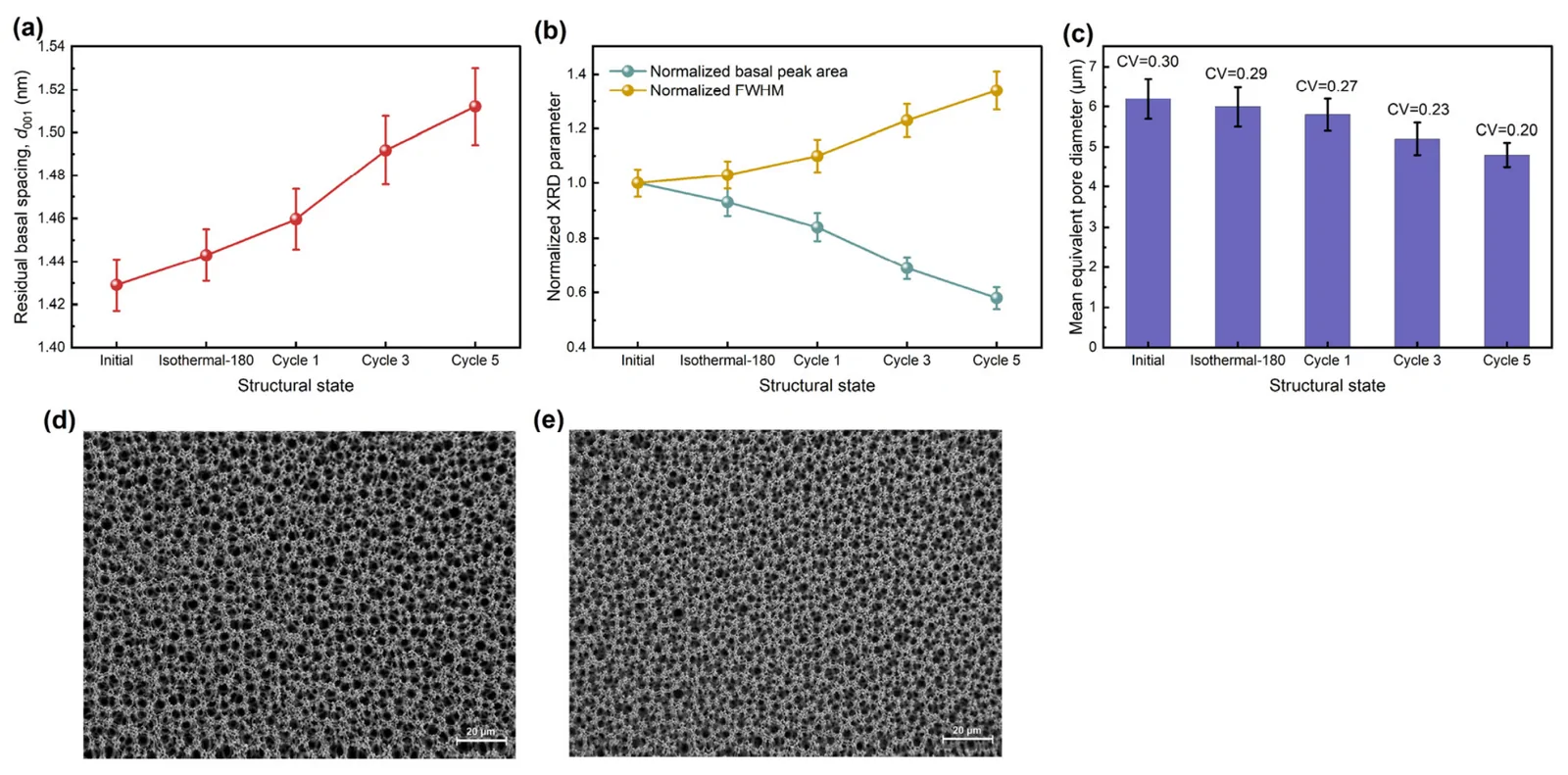
Multiscale structural evolution of Lap-DN during cyclic thermal exposure. (**a**) Evolution of the apparent residual Laponite basal spacing (*d*_001_) measured in identically freeze-dried specimens after continuous and cyclic thermal treatments. (**b**) Normalized area and FWHM of the residual low-angle basal reflection relative to the initial Lap-DN state. The XRD parameters represent comparative ex situ descriptors of residual ordered domains and are not interpreted as direct measurements of the native hydrated platelet structure. (**c**) Mean equivalent pore diameter and corresponding pore-size coefficient of variation after different thermal histories. (**d**) Representative cryo-SEM image of Lap-DN after continuous exposure at 180 °C with the same cumulative high-temperature duration as the cyclic treatment. (**e**) Representative cryo-SEM image of Lap-DN after five thermal cycles. The initial Lap-DN morphology is shown in Figure 5e. All samples used for pore-structure comparison were re-equilibrated to the same hydration condition before cryogenic fixation. As with the rheological measurements, re-equilibration may permit partial structural relaxation; therefore, the cryo-SEM images represent hydration-normalized post-treatment morphologies rather than the instantaneous pore structure existing at the end of the 180 °C exposure. Error bars in (**a**–**c**) represent standard deviations from independently prepared samples.

The cycle-dependent rheological and mechanical reinforcement was accompanied by a progressive change in the structural state of the Laponite-containing network. In the initial Lap-DN, the weak residual basal reflection identified previously corresponded to *d*_001_ = 1.429 nm. Continuous exposure at 180 °C produced only a small increase to 1.443 nm, whereas repeated thermal cycling resulted in a more pronounced shift of the residual reflection toward lower 2*θ* values. The calculated *d*_001_ increased to 1.460 nm after the first cycle, 1.492 nm after the third cycle, and 1.512 nm after the fifth cycle (Figure 10a).

This increase should not be interpreted as uniform expansion of all Laponite platelets within the hydrogel. The low-angle reflection originates only from the fraction of platelets that retains sufficient stacking order to diffract coherently. The evolution of this residual ordered fraction was therefore examined together with the peak area and width. Relative to the initial state, the normalized basal-peak area remained at 0.93 after continuous 180 °C treatment but decreased to 0.84, 0.69, and 0.58 after 1, 3, and 5 thermal cycles, respectively (Figure 10b). In parallel, the normalized FWHM increased from 1.00 initially to only 1.03 after isothermal treatment but reached 1.10, 1.23, and 1.34 after successive cyclic treatments. Within the identically freeze-dried specimens, the combination of decreasing peak area and increasing peak width is consistent with a progressive reduction or redistribution of residual coherent layer ordering. Because the measured reflection is weak and may also be influenced by freeze-drying-induced dehydration, restacking, collapse, and specimen orientation, these changes are treated as comparative ex situ structural fingerprints rather than as unique evidence of in situ platelet rearrangement. Importantly, the magnitude of the XRD change was substantially greater after cyclic treatment than after continuous heating with the same cumulative high-temperature exposure, indicating that the post-treatment dried states retained a reproducible dependence on thermal history.

The micron-scale pore architecture evolved in the same direction, although it reflects a different structural level. The initial Lap-DN morphology presented in Figure 5e had a mean equivalent pore diameter of 6.2 μm and a pore-size coefficient of variation of 0.30. After the isothermal-180 treatment, the mean pore diameter remained close to the initial value at 6.0 μm, with a CV of 0.29 (Figure 10c,d). The limited difference between these two states indicates that cumulative exposure at 180 °C alone produces only minor restructuring once hydration is normalized.

A clearer change developed after repeated heating–cooling cycles. The mean pore diameter decreased to 5.8 μm after the first cycle, 5.2 μm after the third cycle, and 4.8 μm after the fifth cycle, corresponding to a reduction of approximately 22.6% relative to the initial network. At the same time, the pore-size CV decreased from 0.30 to 0.20. The cycle-5 morphology therefore exhibited fewer large pores and a more uniform pore distribution than the initial Lap-DN network, while retaining the same overall interconnected network character (Figure 10e). The change is better described as network refinement and redistribution than as formation of an entirely new morphology.

Within this evidence-limited interpretation, a plausible molecular pathway can be proposed for the thermal-history dependence of the residual Laponite ordering. Heating to 180 °C increases the conformational mobility of the P(NaAMPS-co-NVP-co-AM) chains and accelerates exchange at polymer–Laponite interfaces. Local chain segments containing NaAMPS-, NVP-, and AM-derived units may therefore access previously less accessible hydrated edge or interlayer regions of the residual tactoids. Such thermally enhanced segmental rearrangement could promote transient expansion of accessible galleries and weaken coherent platelet stacking, while the permanent MBAA-crosslinked scaffold limits large-scale polymer displacement. During subsequent cooling and rehydration, the redistributed polymer segments and Laponite-associated constraints may reorganize into a different local configuration, so that repeated thermal transitions progressively favor a lower fraction of coherently stacked domains. The AM- and NVP-derived carbonyl/amide functionalities and the strongly hydrated NaAMPS-derived sulfonate groups may contribute differently to this interfacial rearrangement; however, the present measurements do not resolve the relative contribution of individual comonomer units or directly demonstrate polymer insertion into the Laponite galleries [26,27,28,29]. Taken together, the XRD and cryo-SEM results show that cyclic thermal history is associated with systematic differences in the post-treatment Laponite-associated ordering and hydrated pore architecture. The pore-network refinement is directly supported by cryo-SEM, whereas the XRD changes are interpreted only as ex situ indicators of altered residual layer ordering. Their parallel evolution is consistent with, but does not uniquely demonstrate, a cycle-induced reorganization of the Laponite-containing network.

This post-cycling structural state is consistent with the increases in *G*′, compressive modulus, *σ*_50_, and dissipated energy reported in the preceding sections. Nevertheless, the present measurements remain endpoint characterizations. In particular, the XRD data describe the residual ordered state after freezing and lyophilization rather than the native hydrated structure at 180 °C or during cooling. They do not directly visualize platelet motion or interfacial-junction exchange during heating and cooling. The sequence of thermal dissociation, rearrangement, and reassociation is therefore treated as a mechanistic interpretation supported by the combined structural and mechanical evidence, rather than as a directly observed molecular process.

#### 2.5.2. Water Retention, Volume Stability, and Salinity Tolerance

**Figure 11:**
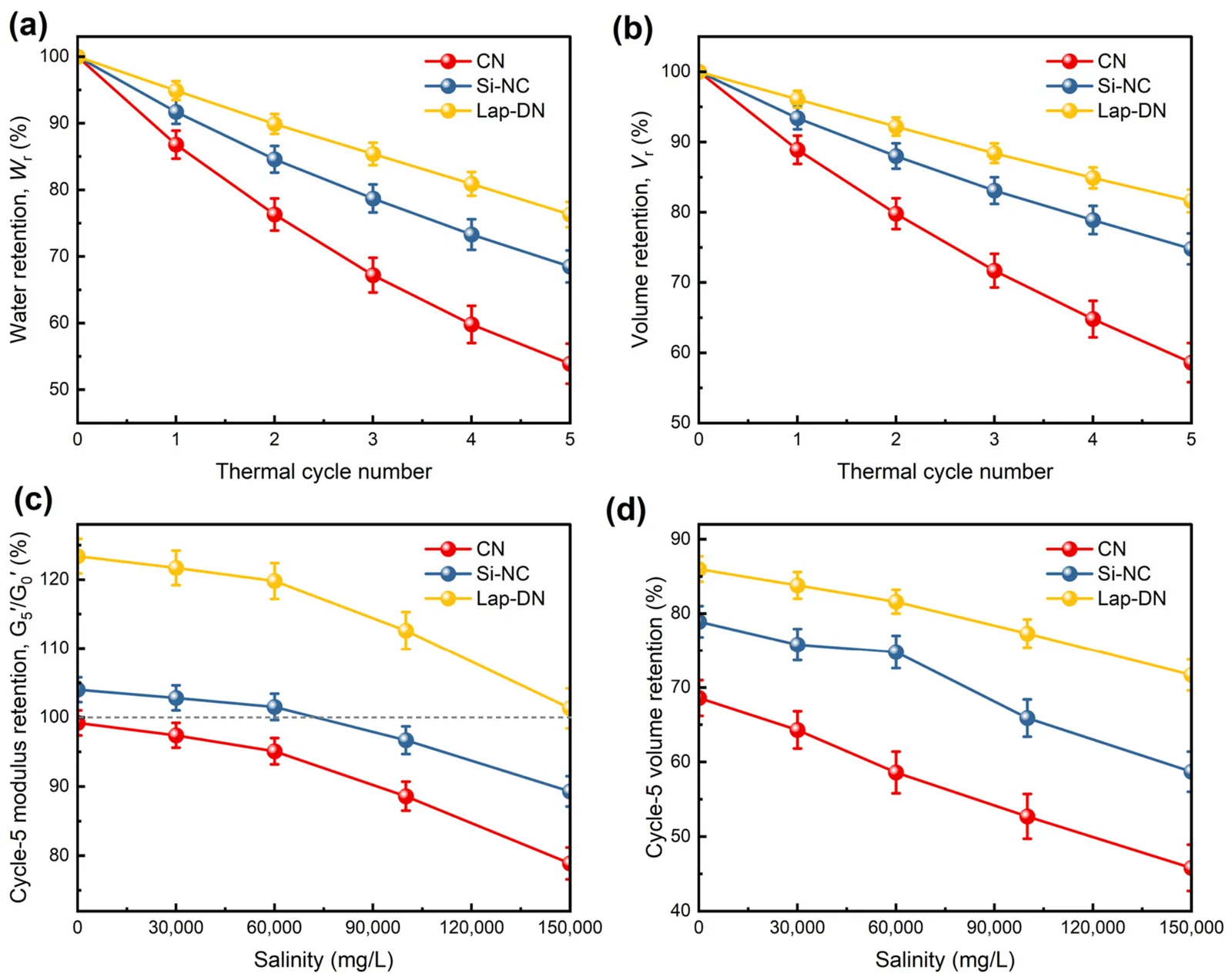
Hydrothermal water retention, volume stability, and salinity tolerance of the hydrogel networks. (**a**) Water-retention ratio (*W*_r_) of CN, Si-NC, and Lap-DN during five thermal cycles at 180 °C in the reference synthetic brine. (**b**) Corresponding volume-retention ratio (*V*_r_) during cyclic thermal exposure. (**c**) Storage-modulus retention after five thermal cycles as a function of salinity; the dashed horizontal line denotes G_5_′/G_0_′ = 100%. (**d**) Cycle-5 volume retention under increasing salinity. Synthetic brines were prepared by proportionally scaling the total dissolved-solids concentration while maintaining the same relative ionic composition. Error bars represent standard deviations from three independently prepared samples.

The structural refinement observed after thermal cycling did not eliminate water loss from the hydrogels, but it substantially reduced its accumulation. Water retention was quantified from the mass of water remaining in the specimen relative to its initial water mass rather than from wet-basis water content alone. This distinction is important because the latter, used in Figure 6a to normalize rheological measurements, does not directly describe the amount of water physically retained by the hydrogel during steam exposure. Under the reference synthetic brine condition, CN retained 86.8% of its initial water after the first cycle and only 53.9% after the fifth cycle (Figure 11a). Si-NC showed a slower decline, reaching 68.5% after five cycles. Lap-DN retained 94.9% after the first cycle and 76.3% after the fifth cycle. The latter value is consistent with the as-recovered water content reported in Section 2.3.1 and confirms that the hydration normalization used for rheological comparison did not conceal the occurrence of partial dehydration during the actual thermal treatment.

The difference among the three networks was also evident from their macroscopic volume stability (Figure 11b). After five cycles, the retained volume decreased to 58.6% for CN and 74.8% for Si-NC, whereas Lap-DN maintained 81.6% of its initial volume. The progressive reduction in both water and volume retention indicates that repeated exposure at 180 °C still imposes a substantial hydrothermal load on the network. The response of Lap-DN is therefore better described as suppressed dehydration and shrinkage, rather than complete resistance to either process. This distinction is particularly relevant to fracture sealing, where severe syneresis can create bypass channels even if the remaining polymer phase retains a high modulus.

Salinity introduced an additional constraint on the cycle-dependent mechanical response. To avoid conflating ionic composition with total dissolved solids, the salinity series was designed by proportionally scaling a fixed synthetic formation-water recipe while maintaining the relative ionic composition. After five thermal cycles, the modulus ratio G_5_′/G_0_′ of Lap-DN decreased from 123.4% in the nominally salt-free condition to 121.7%, 119.8%, 112.6%, and 101.3% at salinities of 30,000, 60,000, 100,000, and 150,000 mg/L, respectively (Figure 11c). The cycle-induced reinforcement therefore remained pronounced up to 100,000 mg/L but became marginal at the highest salinity. Si-NC showed only limited reinforcement at low salinity and fell below its initial modulus above approximately 60,000–100,000 mg/L. CN was progressively weakened as salinity increased, with G_5_′/G_0_′ decreasing to 78.9% at 150,000 mg/L.

The progressive attenuation of cycle-induced reinforcement with increasing salinity is likely governed by coupled changes in Laponite interfacial electrostatics and polymer hydration. Increasing ionic strength compresses the diffuse electrical layer surrounding the negatively charged Laponite surfaces and screens platelet–platelet and polymer–particle electrostatic interactions. Previous studies have shown that inorganic salts weaken the rheological contribution of Laponite in polymer hydrogels and that Ca^2+^ can alter Laponite particle association more strongly than Na^+^ at comparable concentrations [18,29,31]. In parallel, ion-induced screening and reduced hydration restrict the conformational freedom of the polymer chains; ionic strength has likewise been reported to suppress swelling in polyacrylamide/Laponite nanocomposite hydrogels [32]. At the polymer–clay interface, accumulation of hydrated counterions, particularly divalent cations, may further reduce the accessibility of charged or hydroxyl-containing Laponite surface sites to carbonyl-containing NVP- and AM-derived segments. Such competitive interfacial occupation and altered hydration could therefore suppress re-establishment of a fraction of the Laponite-mediated physical constraints during cooling and re-equilibration, thereby reducing the magnitude of thermal self-reinforcement. However, the present experiments do not directly demonstrate competitive occupation of specific carbonyl–Laponite coordination sites, and the salinity series proportionally varied all constituent salts rather than individual ion species. The observed attenuation is therefore interpreted as the combined result of ionic screening, altered interfacial association, and reduced polymer hydration rather than being assigned to a single ion-specific mechanism.

The same ranking was observed in volume stability after five cycles (Figure 11d). At the reference salinity of 60,000 mg/L, the cycle-5 volume retentions of CN, Si-NC, and Lap-DN were 58.6%, 74.8%, and 81.6%, respectively. Increasing the salinity to 150,000 mg/L reduced these values to 45.8%, 58.7%, and 71.7%. Even under this severe condition, Lap-DN therefore retained more than two-thirds of its initial volume, although the marked decline relative to the low-salinity state shows that salinity remains an important limitation rather than a negligible environmental variable.

Taken together, the hydrothermal and salinity results define a laboratory-scale stability envelope for the reinforced network rather than establishing field applicability. Thermal cycling promotes structural and mechanical reinforcement in Lap-DN, but this benefit coexists with progressive water loss and is gradually attenuated as salinity increases. Importantly, mechanical retention and dimensional stability did not evolve identically. At 150,000 mg/L, Lap-DN retained 101.3% of its initial storage modulus after five cycles, whereas its volume retention decreased to 71.7%, corresponding to a 28.3% loss of the initial volume. Thus, retention of bulk mechanical stiffness should not be interpreted as preservation of fracture occupancy or sealing integrity. Such shrinkage may generate interfacial gaps or connected bypass pathways even when the remaining hydrogel matrix retains substantial mechanical strength. Accordingly, the present results demonstrate improved hydrothermal property retention of Lap-DN relative to CN and Si-NC under the controlled laboratory conditions, but they do not establish equivalent sealing performance under all high-salinity reservoir conditions. Because the fracture-sealing experiments in Section 2.6 and Section 2.7 were conducted in the 60,000 mg/L reference brine, the bulk mechanical retention measured at 150,000 mg/L should not be extrapolated directly to fracture-sealing performance at that salinity.

### 2.6. Fracture Sealing Under Steam Exposure

**Figure 12:**
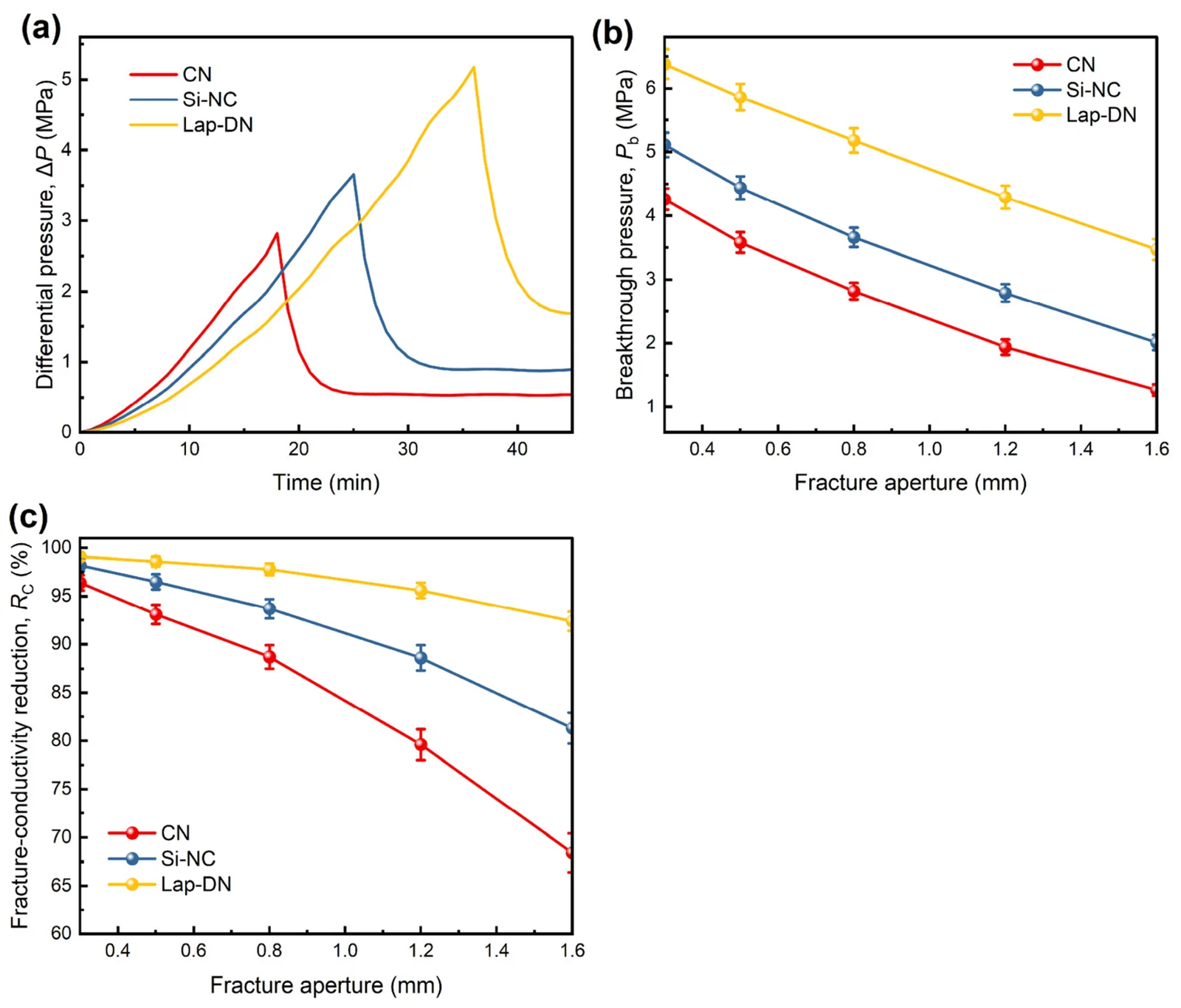
Fracture-sealing performance of the hydrogel networks after steam exposure. (**a**) Representative differential-pressure histories of CN, Si-NC, and Lap-DN in a 0.80 mm fracture after a single 30 min steam exposure at 180 °C. (**b**) Breakthrough pressure (*P*_b_) as a function of fracture aperture. (**c**) Fracture-conductivity reduction (*R*_C_) calculated from paired non-destructive low-differential-pressure conductivity measurements before gel placement and after establishment of the steam-exposed seal across the investigated aperture range. The reference synthetic brine contained 60,000 mg/L total dissolved solids. Error bars in (**b**,**c**) represent standard deviations from three independently prepared sealing specimens.

The material-level reinforcement observed during thermal cycling translated into a distinct improvement in fracture pressure resistance after steam exposure. Representative pressure histories for a 0.80 mm fracture are shown in Figure 12a. Under otherwise identical conditions, CN developed a maximum differential pressure of 2.82 MPa before breakthrough, while Si-NC sustained 3.66 MPa. Lap-DN continued to accumulate pressure to 5.18 MPa before the first sustained downstream breakthrough. The subsequent pressure drop confirms that the recorded maximum reflects failure of the established seal rather than continued compression of a closed hydraulic system. The later breakthrough observed for Lap-DN is consistent with the higher pressure resistance of the sealing body, although breakthrough time itself is not treated as an independent performance metric because it also depends on system compliance and the imposed flow condition.

The influence of fracture aperture was more revealing (Figure 12b). Breakthrough pressure decreased for all three materials as the aperture increased from 0.30 to 1.60 mm, reflecting the progressively larger unsupported span and greater flow capacity of the open fracture. For CN, *P*_b_ decreased from 4.26 to 1.27 MPa over this range. Si-NC performed better, retaining 2.01 MPa at 1.60 mm. Lap-DN showed both a higher absolute pressure resistance and a smaller fractional loss with increasing aperture: *P*_b_ decreased from 6.38 MPa at 0.30 mm to 5.18 MPa at 0.80 mm and remained at 3.47 MPa even at 1.60 mm. At the representative aperture of 0.80 mm, the breakthrough pressure of Lap-DN was approximately 83.7% higher than that of CN and 41.5% higher than that of Si-NC.

This difference became more pronounced toward the wide-fracture end of the series. At 1.60 mm, the breakthrough pressure of Lap-DN was approximately 2.7 times that of CN. The result is consistent with the finite-strain mechanical behavior established in Section 2.4: a sealing body spanning a wider fracture experiences larger local deformation before pressure failure, so the benefit of the higher *σ*_50_, compressive modulus, and post-deformation recovery of Lap-DN becomes increasingly relevant as aperture increases. The sealing data therefore provide a functional counterpart to the mechanical measurements rather than an isolated engineering metric.

Breakthrough pressure alone does not describe the degree to which the original high-conductivity pathway is suppressed. The fracture-conductivity reduction

(5) $$R_{C}\;=\;\left(1\;-\;\frac{C_{f}}{C_{f,0}}\right)\;\times \;100\%$$

was therefore evaluated independently, where *C*_f__,0_ and *C*_f_ denote the fracture conductivity before gel placement and after formation of the steam-exposed seal, respectively. At 0.30 mm aperture, all three systems produced substantial conductivity reductions, exceeding 96% (Figure 12c). Their responses diverged as the fracture became wider. At 1.20 mm, *R*_C_ decreased to 79.6% for CN and 88.6% for Si-NC, whereas Lap-DN remained at 95.6%. Even at 1.60 mm, Lap-DN reduced fracture conductivity by 92.4%, compared with 81.3% for Si-NC and 68.4% for CN.

The combination of high breakthrough pressure and low residual conductivity is important because the two quantities describe different failure modes. A seal may temporarily sustain a large pressure yet still leave connected bypass pathways, while a low-conductivity seal may fail mechanically at a relatively small differential pressure. Lap-DN retained both attributes over the investigated aperture range. In the context of the preceding structural and mechanical results, this behavior is consistent with a sealing body that remains highly hydrated and mechanically coherent after steam exposure while accommodating the deformation imposed by increasingly wide fractures. Whether this integrity can be recovered repeatedly after successive steam-induced failures is examined in the following cyclic resealing experiments.

The present fracture-flow experiments should nevertheless be interpreted as controlled material-level comparisons rather than direct replicas of natural reservoir fractures. The nominal fracture aperture was defined geometrically so that the effect of aperture could be examined independently, whereas natural fractures exhibit coupled variations in surface roughness, local aperture, mineral composition, and fluid conditioning. Surface roughness may increase the real contact area and mechanical interlocking of a hydrogel seal, but it can also create preferential grooves, local stress concentrations, and nonuniform load transfer. Likewise, spatially heterogeneous apertures may promote local bridging at constrictions while simultaneously preserving wider connected flow pathways that impose greater deformation on the sealing body. Therefore, the absolute breakthrough pressures and conductivity reductions measured here should not be transferred directly to reservoir-scale fractures without accounting for fracture-topology effects.

Interfacial chemistry introduces an additional source of uncertainty. Quartz-, carbonate-, and clay-rich fracture surfaces can differ substantially in wettability, surface charge, and polymer adsorption behavior, which may modify hydrogel adhesion, penetration, and mechanical anchoring at the fracture wall. Prior exposure to heavy crude oil may further alter these interactions by forming oil- or polar-component-rich surface films, changing the local wettability and reducing direct contact between the aqueous hydrogel and the mineral surface. Under steam conditions, redistribution of these oil films and condensate may introduce additional time-dependent interfacial effects. These factors were not isolated in the present laboratory model; consequently, the results establish the intrinsic comparative sealing and resealing capability of the three hydrogel systems under controlled geometry, whereas reservoir-scale performance will require validation using rough, mineralogically heterogeneous, nonuniform fractures under crude-oil/steam exposure.

### 2.7. Cyclic Steam Impact–Recovery–Resealing Performance

A cyclic sealing experiment was designed to distinguish genuine resealing from repeated tests performed with newly prepared specimens. Each sealing body remained in the same 1.20 mm fracture throughout five consecutive steam impact–recovery cycles. During each cycle, the established seal was challenged at 180 °C until sustained downstream breakthrough occurred. The system was then depressurized and cooled at 25 °C for 30 min without adding or repositioning hydrogel, after which the same sealing body was reheated and challenged again.

**Figure 13:**
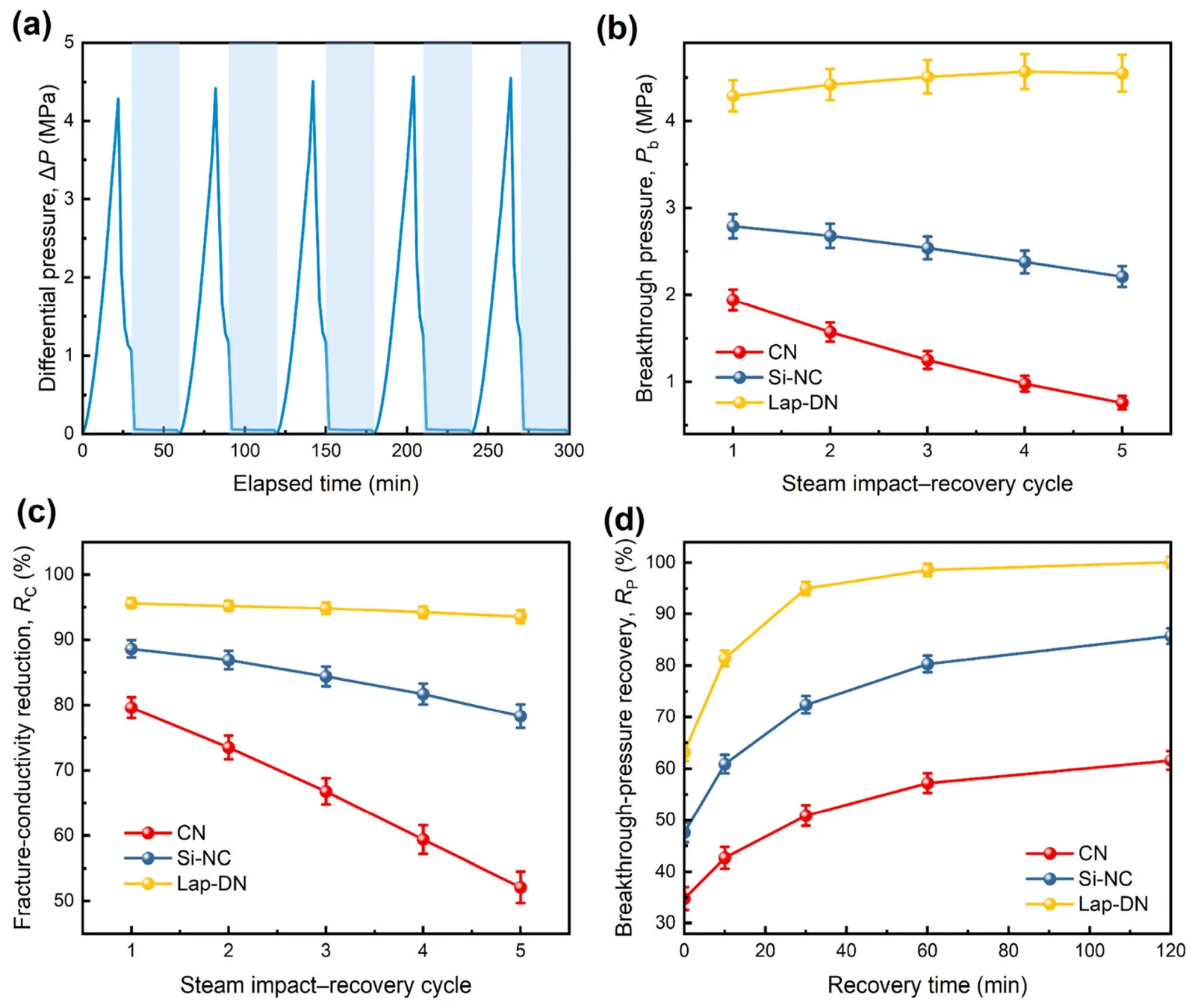
Cyclic steam impact–recovery–resealing performance of the hydrogel networks. (**a**) Continuous differential-pressure history of the same Lap-DN sealing body during five consecutive steam impact–recovery cycles in a 1.20 mm fracture. Each cycle consisted of a 30 min high-temperature challenge at 180 °C followed by 30 min cooling and recovery at 25 °C; no additional hydrogel was introduced between cycles. (**b**) Breakthrough pressure (*P*_b_) of CN, Si-NC, and Lap-DN over five consecutive cycles. (**c**) Fracture-conductivity reduction (*R*_C_) after each resealing cycle. (**d**) Recovery of breakthrough-pressure resistance (*R*_P_) as a function of resting time after a standardized fifth-cycle breakthrough. The reference brine contained 60,000 mg/L total dissolved solids. Error bars in (**b**–**d**) represent standard deviations from three independently prepared sealing specimens.

The continuous pressure history of Lap-DN illustrates the repeated failure–recovery sequence directly (Figure 13a). During the first steam challenge, the differential pressure increased to 4.29 MPa before breakthrough, consistent with the single-exposure result obtained at the same aperture in Figure 12. After pressure release and a 30 min recovery period, the seal re-established sufficient integrity to withstand 4.42 MPa during the second challenge. The breakthrough pressure increased further to 4.51 and 4.57 MPa during cycles 3 and 4 and remained at 4.55 MPa during the fifth cycle. Each breakthrough was followed by an abrupt pressure decrease, confirming that the subsequent pressure accumulation originated from re-establishment of the sealing structure rather than uninterrupted pressure storage in the apparatus.

The three hydrogel systems showed markedly different cumulative responses (Figure 13b). CN lost pressure resistance progressively, with *P*_b_ decreasing from 1.94 MPa in the first cycle to 0.76 MPa in the fifth cycle. Si-NC exhibited a slower decline, from 2.79 to 2.21 MPa. Lap-DN did not follow either trend. Its breakthrough pressure increased slightly during the first four cycles and remained 4.55 MPa after the fifth cycle, corresponding to 106.1% of the first-cycle value. The modest magnitude of this increase is noteworthy. Although the material-level measurements showed substantial thermal self-reinforcement, repeated fracture breakthrough simultaneously introduces mechanical damage and local flow-channel formation. The sealing response therefore reflects competition between cycle-induced reinforcement and accumulated damage rather than a monotonic transfer of the full mechanical-strength increase into *P*_b_.

The ability to suppress fracture conductivity showed the same distinction, although it did not increase with cycle number (Figure 13c). Lap-DN reduced the fracture conductivity by 95.6% after the first sealing cycle and retained a reduction of 93.5% after the fifth cycle. Thus, approximately 97.8% of its initial conductivity-reduction capability was preserved after four additional breakthrough–recovery events. Si-NC decreased from 88.6% to 78.3%, whereas CN declined from 79.6% to 52.1%. The slight deterioration in *R*_C_ for Lap-DN, despite the maintained breakthrough pressure, suggests that small residual flow pathways accumulate during repeated failure even when the load-bearing sealing structure is largely restored. This distinction prevents the cyclic response from being interpreted as perfect self-healing.

The recovery interval required for resealing was examined separately after a standardized fifth-cycle breakthrough (Figure 13d). Immediately after pressure release, Lap-DN recovered 63.2% of its pre-breakthrough pressure resistance. The recovery ratio increased rapidly to 81.4% after 10 min and 95.0% after 30 min, reaching 98.6% after 60 min and approximately 100% after 120 min. Si-NC showed a slower and incomplete recovery, reaching 85.7% after 120 min, while CN recovered only 61.6%. The 30 min recovery interval used in the cyclic experiment therefore captured most of the functional recovery of Lap-DN without requiring complete equilibration.

The cyclic fracture experiment establishes a functional consequence of the thermally responsive network that cannot be inferred from rheology or compression alone. Lap-DN retains high pressure resistance after repeated breakthrough, rapidly restores sealing capability during cooling, and maintains a fracture-conductivity reduction above 93% after five cycles without replenishment of the sealing material. The small loss in conductivity control alongside the nearly preserved breakthrough pressure also shows that resealing is substantial but not idealized. This balance between recovery and accumulated damage provides a more realistic basis for interpreting repeated fracture sealing under steam-service conditions.

### 2.8. Structure–Property–Sealing Relationship and Proposed Thermal Self-Reinforcement Model

**Figure 14:**
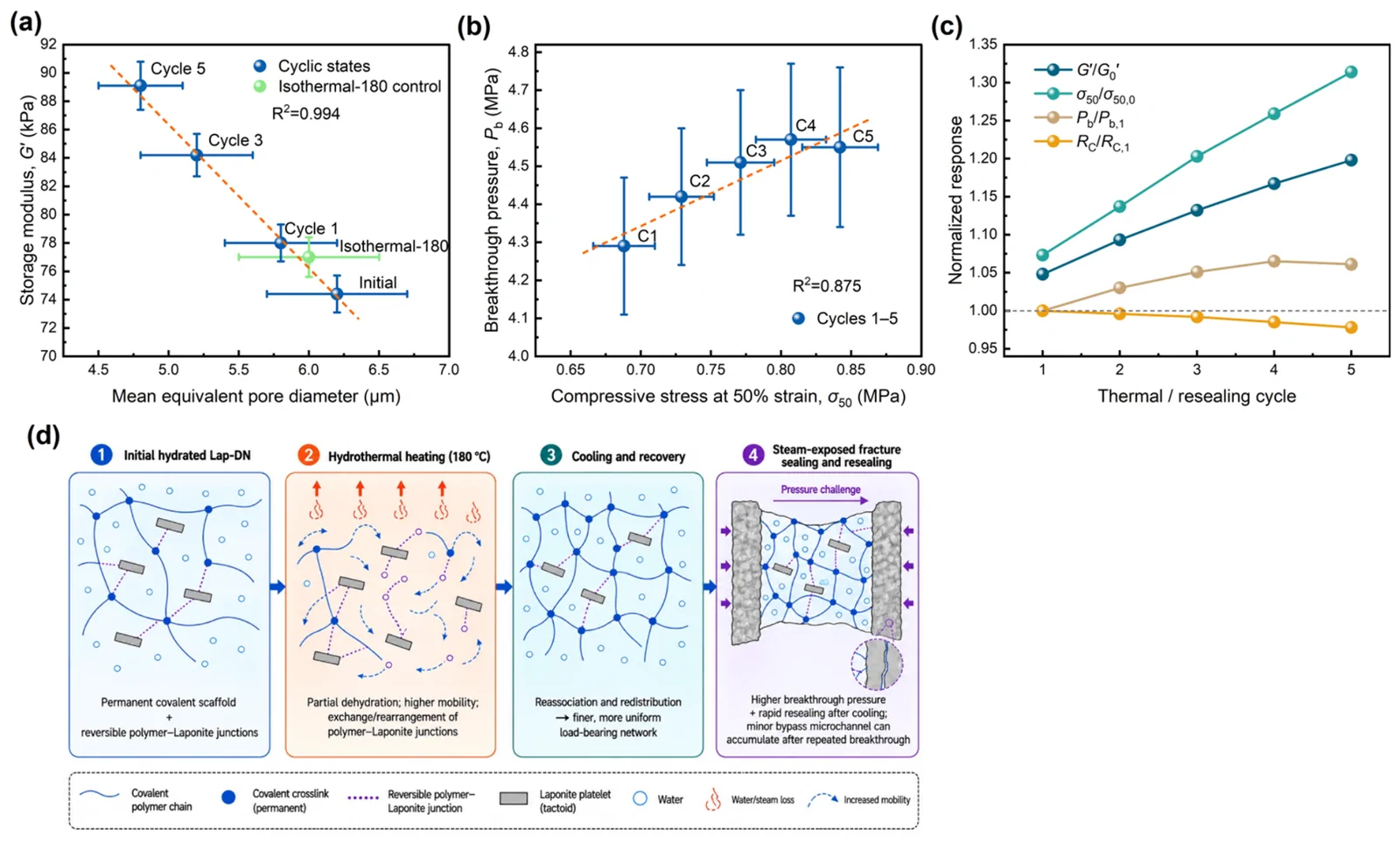
Structure–property–sealing relationships and proposed thermal self-reinforcement model of Lap-DN. (**a**) Relationship between mean equivalent pore diameter and storage modulus (*G*′) for the initial, isothermal-180, and cyclically treated Lap-DN states. The cyclic states are connected to indicate their progressive structural evolution, while the isothermal-180 sample is shown as a parallel thermal-dose control. (**b**) Relationship between compressive stress at 50% strain (*σ*_50_) and breakthrough pressure (*P*_b_) in a 1.20 mm fracture over cycles 1–5. (**c**) Normalized evolution of rheological reinforcement, compressive reinforcement, breakthrough-pressure retention, and fracture-conductivity reduction during repeated thermal/resealing cycles. (**d**) Conceptual structure–property model linking cyclic thermal perturbation, the proposed reorganization of Laponite-mediated physical constraints, mechanical self-reinforcement, and repeated fracture resealing. Error bars in (**a**,**b**) represent standard deviations from three independent replicate measurements. The dashed orange lines in (**a**,**b**) represent descriptive linear regression fits to the corresponding cyclic-state data, with the R^2^ values shown only as descriptive goodness-of-fit indicators and not for inferential statistical analysis. In (**a**), the isothermal-180 control was not included in the regression fit.

The structural, mechanical, and sealing responses developed coherently during cyclic thermal exposure, but their magnitudes were not identical. The most direct structure–property relationship was observed between pore-network refinement and the small-deformation elastic response of Lap-DN. The mean equivalent pore diameter decreased from 6.2 μm in the initial state to 5.8, 5.2, and 4.8 μm after 1, 3, and 5 thermal cycles, respectively, while *G*′ increased from 74.4 to 78.0, 84.2, and 89.1 kPa (Figure 14a). The isothermal-180 control remained close to the initial condition, with a pore diameter of 6.0 μm and *G*′ = 77.0 kPa. The divergence between continuous and cyclic heating indicates that the finer network developed during repeated heating–cooling transitions rather than simply through cumulative exposure at 180 °C.

The same tendency extended from the small-strain rheological scale to finite-strain mechanical loading. Across cycles 1–5, the compressive stress at 50% strain of Lap-DN increased from 0.688 to 0.842 MPa, while the breakthrough pressure in a 1.20 mm fracture increased from 4.29 to approximately 4.55 MPa (Figure 14b). The relationship was not proportional over the full range. *P*_b_ increased during the first four cycles but approached a plateau by the fifth cycle even though *σ*_50_ continued to rise. This deviation is physically meaningful because fracture sealing is controlled not only by the strength of the hydrogel but also by local interface deformation, accumulated bypass channels, and damage created during preceding breakthrough events.

The different rates of change become clearer when the principal responses are normalized to their respective reference states (Figure 14c). After five thermal cycles, Lap-DN reached

(6) $$\frac{G_{5}^{\prime }}{G_{0}^{\prime }}=1.198$$

and

(7) $$\frac{\sigma_{50,5}}{\sigma_{50,0}}=1.314$$

corresponding to increases of approximately 19.8% and 31.4%, respectively. In contrast, the fifth-cycle breakthrough pressure was 106.1% of the first-cycle value, while the fracture-conductivity reduction retained 97.8% of its first-cycle level. The mechanical state of the hydrogel therefore strengthened more strongly than the macroscopic sealing response. Repeated failure simultaneously generates local damage, so only part of the material-level reinforcement is converted into additional pressure resistance. The nearly preserved *R*_C_, rather than an unrealistic increase in every sealing metric, is consistent with a sealing body that progressively strengthens while accumulating a small amount of irreversible flow-path damage.

To place the present performance in the context of existing reservoir gel technologies, representative high-temperature conformance-control gels reported in the recent literature are summarized in Table 1. Because gel format, swelling state, brine chemistry, test geometry, and performance criteria differ substantially among studies, these values are intended as contextual benchmarks rather than as a strict numerical ranking. High-temperature PPGs have demonstrated exceptional long-term hydrothermal stability at 150 °C [10], whereas self-healing Alg-IPNGs and re-crosslinkable PPGs have demonstrated structural recovery at approximately 110–130 °C [11,23]. Chitosan-grafted PPGs have additionally shown strong tolerance to high-temperature and high-salinity environments [19], while Laponite-containing chemically crosslinked hydrogels have demonstrated rapid rheological reconstruction but with thixotropic performance that becomes less favorable as temperature and salinity increase [18]. In comparison, the distinctive feature of Lap-DN is the simultaneous combination of repeated exposure at 180 °C, cycle-induced mechanical reinforcement, and rapid functional resealing of the same fracture-confined hydrogel body after breakthrough. The present system is therefore not considered universally superior to previously reported gels; rather, it represents a complementary materials-design strategy in which cyclic thermal perturbation itself is exploited to reorganize the reversible physical network and sustain repeated fracture sealing.

The combined evidence is consistent with a proposed thermally triggered network-reorganization model in which the two populations of network constraints play distinct roles (Figure 14d). In the initial hydrated state, the MBAA-crosslinked P(NaAMPS-co-NVP-co-AM) network provides permanent macroscopic connectivity, while Laponite platelets introduce reversible polymer–particle constraints. Hydrothermal heating at 180 °C was accompanied by partial dehydration, while the post-treatment XRD response showed a decrease in the normalized basal-peak area together with peak broadening, consistent with reduced coherent stacking within the residual Laponite tactoid population. These endpoint observations do not resolve the real-time motion of individual platelets or the exchange kinetics of individual polymer–Laponite junctions. Accordingly, increased segmental mobility and partial weakening, exchange, or redistribution of polymer–Laponite associations during heating are proposed as plausible molecular-level processes rather than treated as directly observed events. Because the permanent covalent scaffold remains intact, such transient rearrangement, if operative, would not require macroscopic collapse of the polymer network.

Within the proposed model, cooling and rehydration could permit re-establishment or redistribution of a portion of the Laponite-mediated physical constraints within the preserved covalent framework. The 24 h standardized re-equilibration used before ex situ characterization may contribute to this recovery-stage reorganization; therefore, the measured final network state reflects the combined consequence of prior thermal history and subsequent controlled recovery rather than the high-temperature stage alone. Repeated heating–cooling transitions may therefore progressively shift the network toward the experimentally observed post-cycling state characterized by smaller and more uniformly distributed pores and a reduced population of coherently stacked tactoid domains. This structural evolution is consistent with the concurrent increases in *G*′, compressive modulus, *σ*_50_, and dissipated energy, together with the retained rapid recovery after large deformation.

When the post-cycling network is confined within a fracture, the combination of permanent covalent connectivity and dynamically recoverable physical constraints is consistent with two experimentally observed functional advantages. First, the refined load-bearing structure is associated with higher differential-pressure resistance before breakthrough. Second, after local seal failure, depressurization and cooling were accompanied by substantial recovery of pressure resistance in the same hydrogel body without addition of fresh material; within the proposed model, this functional recovery is attributed in part to reorganization of the Laponite-mediated physical constraints. The slight decline in conductivity reduction over repeated breakthrough cycles shows that the process is not perfectly reversible, but the retained pressure resistance and rapid resealing demonstrate that the post-cycling network remains functionally effective.

This model should be interpreted as a proposed structure–property framework rather than an in situ molecular observation. The structural measurements were obtained from post-treatment states and therefore do not directly visualize the transient dissociation and reassociation of individual polymer–Laponite junctions during heating and cooling. The progressive loss of residual platelet stacking, pore-network refinement, hydration-normalized reinforcement, dynamic recovery, and repeated resealing nevertheless provide mutually consistent evidence for a cycle-induced reorganization of the physical network within the permanent covalent scaffold. The attenuation of both reinforcement and volume retention at high salinity further indicates that the proposed reorganization process remains sensitive to ionic screening and hydration state rather than being an irreversible secondary crosslinking process. The sealing component of this structure–property model is likewise limited to the controlled laboratory fracture geometry used here and should not be interpreted as a quantitative prediction for rough, mineralogically heterogeneous, oil-conditioned reservoir fractures.

## 3. Conclusions

A thermally triggered self-reinforcing covalent–physical double-network nanocomposite hydrogel was developed by integrating a sparse MBAA-crosslinked P(NaAMPS-co-NVP-co-AM) covalent scaffold with a Laponite-mediated reversible physical network for repeated fracture sealing under cyclic steam exposure. Screening of the covalent-network composition and nanoclay content identified a NaAMPS/NVP/AM molar ratio of 40/20/40 and a Laponite loading of 1.00 wt% as the working formulation, providing a balance among hydrothermal stability, gelation kinetics, precursor flowability, and mature network stiffness. FTIR showed only a subtle perturbation of the local polymer environment, while XPS verified incorporation of the Laponite-derived inorganic phase and XRD, TEM, and cryo-SEM characterized the associated multiscale structural state. Artifact-controlled comparisons further showed that a persistent cycle-dependent reinforcement remained after standardized hydration normalization and comparison with the corresponding control thermal histories. After five thermal cycles, the storage modulus of Lap-DN increased from 74.4 to 89.1 kPa, while the compressive stress at 50% strain increased from 0.641 to 0.842 MPa, corresponding to reinforcement ratios of 1.198 and 1.314, respectively. Concurrently, the mean equivalent pore diameter decreased from 6.2 to 4.8 μm and the pore-size coefficient of variation decreased from 0.30 to 0.20, accompanied by progressive broadening and weakening of the residual Laponite basal reflection. These observations are consistent with cycle-induced reorganization of the Laponite-mediated physical network within the permanent covalent scaffold. Because the structural measurements represent post-treatment states, reversible weakening, exchange, and reassociation of individual polymer–Laponite junctions are proposed as a plausible molecular-level interpretation rather than claimed as directly observed processes. Under the controlled laboratory conditions, Lap-DN exhibited greater hydrothermal property retention than the two reference systems, retaining 76.3% of its initial water and 81.6% of its initial volume after five cycles in the reference brine, and maintained a cycle-5 modulus retention of 101.3% even at 150,000 mg/L salinity. However, its volume retention decreased to 71.7% at 150,000 mg/L, demonstrating that mechanical retention alone does not ensure dimensional or sealing stability under severe salinity. Under the reference-brine condition, the reinforced network was also associated with improved fracture-sealing performance. Lap-DN sustained breakthrough pressures of 5.18 MPa in a 0.80 mm fracture and 3.47 MPa in a 1.60 mm fracture, while retaining a fracture-conductivity reduction of 92.4% at the largest aperture. In a 1.20 mm fracture, the same sealing body maintained a breakthrough pressure of 4.55 MPa after five steam impact–recovery cycles, equivalent to 106.1% of the first-cycle value, while preserving 93.5% fracture-conductivity reduction. Following breakthrough, approximately 95.0% of the pressure resistance was restored within 30 min of cooling. These findings establish a coherent structure–property–sealing correlation among cycle-induced network reorganization, mechanical self-reinforcement, and repeated fracture resealing, providing a materials-design strategy for hydrogel sealing under steam-intensive thermal-recovery conditions. The proposed mechanism is presently based on post-treatment structural and macroscopic property measurements rather than direct in situ observation of polymer–Laponite junction rearrangement. Future work should therefore examine longer-term cycling, in situ structural evolution, complex field brines, crude-oil-conditioned interfaces, spatially heterogeneous fracture apertures, and rough, mineralogically representative fracture surfaces to further assess the durability and field applicability of the system.

## 4. Materials and Methods

### 4.1. Materials and Instruments

#### 4.1.1. Materials

Acrylamide (AM, ≥99%), sodium 2-acrylamido-2-methylpropane sulfonate (NaAMPS, ≥98%), N-vinyl-2-pyrrolidone (NVP, ≥99%), N,N′-methylenebisacrylamide (MBAA, ≥99%), ammonium persulfate (APS, ≥98%), and hydrophilic nano-SiO^2^ (nominal particle size of approximately 20–30 nm) were obtained from Shanghai Aladdin Bio-Chem Technology Co., Ltd. (Shanghai, China). Laponite XLG was supplied by BYK-Chemie GmbH (Wesel, Germany).

Sodium chloride (NaCl, ≥99.5%), potassium chloride (KCl, ≥99.5%), calcium chloride (CaCl_2_, ≥96.0%), magnesium chloride hexahydrate (MgCl_2_·6H_2_O, ≥98.0%), and sodium sulfate (Na_2_SO_4_, ≥99.0%) were of analytical grade and obtained from Sinopharm Chemical Reagent Co., Ltd. (Shanghai, China). High-purity nitrogen (99.999%) was used for deoxygenation of the precursor solutions. Ultrapure water with a resistivity of 18.2 MΩ·cm was used throughout the experiments. All chemicals were used as received without further purification. The reference synthetic brine used throughout the hydrothermal and fracture-sealing experiments had a nominal total dissolved-solids concentration of 60,000 mg/L. Its detailed salt composition is provided in Table 2. Brines with other salinities were prepared by proportionally scaling all constituent salts while maintaining the same relative salt composition.

#### 4.1.2. Instruments

Rheological measurements were performed using an MCR 302e rotational rheometer (Anton Paar GmbH, Graz, Austria). Fourier-transform infrared spectra were collected using a Nicolet iS50 FTIR spectrometer (Thermo Fisher Scientific, Waltham, MA, USA), while X-ray photoelectron spectroscopy was conducted using a K-Alpha XPS system (Thermo Fisher Scientific, East Grinstead, UK). X-ray diffraction patterns were obtained using a SmartLab X-ray diffractometer (Rigaku Corporation, Tokyo, Japan) equipped with Cu Kα radiation.

Transmission electron microscopy was performed using a JEM-F200 field-emission transmission electron microscope (JEOL Ltd., Tokyo, Japan), and hydrated pore morphology was characterized using a Quattro S field-emission scanning electron microscope equipped with a PP3010 cryogenic preparation system (Thermo Fisher Scientific/Quorum Technologies, Lewes, UK). Mechanical compression and loading–unloading tests were performed using an Instron 5944 universal testing system (Instron, Norwood, MA, USA) equipped with a 100 N load cell.

Hydrothermal and cyclic thermal treatments were conducted using a Series 4560 high-pressure reactor (Parr Instrument Company, Moline, IL, USA). Residual monomer contents were determined using an Agilent 1260 Infinity II high-performance liquid chromatography system (Agilent Technologies, Santa Clara, CA, USA). Fracture-sealing experiments were conducted using a custom-built high-temperature variable-aperture fracture-flow system equipped with a high-pressure metering pump, upstream and downstream pressure transducers, a downstream back-pressure regulator, temperature-control components, and a computerized data-acquisition unit.

### 4.2. Preparation of the Covalent–Physical Nanocomposite Hydrogels

The three principal hydrogel systems used in the subsequent experiments were prepared from the same P(NaAMPS-co-NVP-co-AM) covalent-network formulation. The NaAMPS/NVP/AM molar ratio was fixed at 40/20/40, corresponding to a total monomer concentration of 20.0 wt% in the final precursor. MBAA was used as the permanent covalent crosslinker at 0.20 wt% relative to the total monomer mass, while APS was used as the thermal initiator at 0.50 wt% relative to the total monomer mass. The detailed mass formulation based on 100 g of precursor is given in Table 3.

Three formulations were defined. CN contained only the MBAA-crosslinked P(NaAMPS-co-NVP-co-AM) network and served as the covalent-network control. Si-NC contained the same covalent network together with 1.00 wt% hydrophilic nano-SiO_2_ and served as a mass-matched inorganic nanoparticle reference formulation. Lap-DN contained 1.00 wt% Laponite XLG and constituted the Laponite-mediated covalent–physical nanocomposite formulation investigated throughout the thermal-cycling and fracture-sealing experiments. The identical 1.00 wt% inorganic loading was selected to maintain the same nominal nanoparticle mass fraction; it was not intended to imply equivalence in particle number, specific surface area, surface chemistry, morphology, or density of polymer-accessible interaction sites between nano-SiO2 and Laponite. Accordingly, Si-NC was used as a practical non-layered nanoparticle reference rather than as a fully physicochemically matched control.

For preparation of Lap-DN, the required amount of Laponite XLG was slowly introduced into approximately two-thirds of the total deionized water under continuous stirring at 1000 r/min for 30 min. The dispersion was then sonicated in an ultrasonic bath for 10 min while the temperature was maintained below 25 °C. AM and NVP were dissolved sequentially in the Laponite dispersion, followed by gradual addition of NaAMPS. NaAMPS was added only after the Laponite had been fully hydrated and dispersed so that the nanosilicate was not exposed prematurely to the higher ionic strength of the complete monomer solution. Stirring was continued until no visible agglomerates remained. Si-NC was prepared using the same procedure, with hydrophilic nano-SiO_2_ replacing Laponite at the same 1.00 wt% loading. CN followed the same mixing sequence without addition of an inorganic nanophase.

MBAA was subsequently introduced from a dilute aqueous stock solution, after which the precursor was purged with nitrogen for 20 min to minimize oxygen inhibition of free-radical polymerization. A freshly prepared APS solution was added immediately before casting, and the precursor was gently mixed for approximately 2 min under nitrogen while minimizing air entrainment. No redox accelerator was employed. The precursor was transferred into sealed molds and thermally polymerized at 70 °C for 12 h. This curing period was substantially longer than the rheologically determined gelation times used in Section 4.3 and was selected to obtain a mature covalent network before any hydrothermal, rheological, mechanical, or fracture-sealing treatment.

After polymerization, the hydrogels were removed from the molds and washed in deionized water for 24 h, with the washing medium replaced every 6 h, to remove readily extractable soluble species. Unless otherwise specified, the washed specimens were subsequently equilibrated in the reference synthetic brine containing 60,000 mg/L total dissolved solids at 25 °C. Equilibration was considered complete when the specimen mass changed by less than 0.5% over a 6 h interval. Bulk hydrogels used for rheological, mechanical, and structural characterization were prepared from independent synthesis batches and equilibrated to the prescribed hydration state before testing. Fresh precursor solutions were used for the in situ fracture-sealing experiments described in Section 4.9 and Section 4.10.

In this study, the designation Lap-DN is used to describe a covalent–physical architecture containing two functionally distinct populations of network constraints rather than two independently polymerized covalent networks. The MBAA-crosslinked P(NaAMPS-co-NVP-co-AM) framework provides permanent macroscopic connectivity, whereas reversible polymer–Laponite associations provide a second population of physical constraints capable of rearrangement under thermal perturbation. These physical constraints develop concurrently with covalent-network formation and therefore do not constitute a separately synthesized polymer network. This definition is used consistently throughout the manuscript when referring to the covalent–physical double-network behavior of Lap-DN.

### 4.3. Formulation Screening and Gelation-Kinetics Measurements

The covalent-network composition was first screened without addition of inorganic nanoparticles. NVP was fixed at 20 mol%, while the NaAMPS/AM ratio was varied to obtain NaAMPS/NVP/AM molar ratios of 20/20/60, 30/20/50, 35/20/45, 40/20/40, 45/20/35, 50/20/30, and 60/20/20. The total monomer concentration, MBAA content, and APS content were maintained at 20.0 wt%, 0.20 wt% of the total monomer mass, and 0.50 wt% of the total monomer mass, respectively. Fully cured specimens were equilibrated in the reference synthetic brine (60,000 mg/L) and subjected to a single hydrothermal treatment at 180 °C for 150 min. After cooling and re-equilibration in fresh reference brine for 24 h, the storage modulus was measured at 25 °C, 1% strain, and 1 rad·s^−1^. Gel fraction was determined from the ratio of the dry mass remaining after 72 h aqueous extraction to the initial dry mass.

Using the selected NaAMPS/NVP/AM ratio of 40/20/40, Laponite XLG contents of 0, 0.25, 0.50, 0.75, 1.00, 1.25, 1.50, and 2.00 wt% of the final precursor mass were subsequently evaluated. Gelation kinetics were measured by oscillatory time sweeps immediately after APS addition using a parallel-plate geometry at 1% strain and 1 rad·s^−1^. The 1.00 wt% Lap-DN precursor was tested at 50, 60, 70, 80, and 90 °C, whereas the Laponite-concentration series was evaluated at 70 °C. A solvent trap was used to minimize water evaporation. The operational gelation time, *t*_gel_, was defined as the first persistent crossover of *G*′ and *G*′′, after which *G*′ remained higher than *G*′′.

Mature storage moduli were measured separately from the gelation time sweeps. Hydrogels containing 0–2.00 wt% Laponite were polymerized at 70 °C for 12 h, washed, equilibrated to the same hydration condition, and tested at 25 °C, 1% strain, and 1 rad·s^−1^. Precursor viscosity was measured at 25 °C and a shear rate of 10 s^−1^ using formulations containing all components except APS to avoid polymerization during the measurement. The mean viscosity over the final 30 s of a 60 s measurement was recorded. Unless otherwise specified, three independently prepared samples were tested for each formulation.

The formulation containing 1.00 wt% Laponite was subsequently used as the standard Lap-DN system.

### 4.4. Baseline Rheological and Physicochemical Characterization

The baseline rheological properties of CN, Si-NC, and Lap-DN were characterized at 25 °C. Precursor flow behavior was measured using formulations containing all components except APS to avoid polymerization during testing. After 5 min of equilibration on the rheometer, the shear rate was increased logarithmically from 0.1 to 1000 s^−1^, and the corresponding apparent viscosity was recorded.

The linear viscoelastic region of the fully cured Lap-DN hydrogel was determined by an oscillatory amplitude sweep at an angular frequency of 1 rad·s^−1^. The strain was increased progressively from the small-deformation region until an appreciable decrease in *G*′ was observed. Based on this test, a strain amplitude of 1% was used for subsequent oscillatory measurements. Frequency sweeps of CN, Si-NC, and Lap-DN were then performed at 25 °C and 1% strain over an angular-frequency range of 0.1–100 rad·s^−1^, and the storage modulus (*G*′) and loss modulus (*G*′′) were recorded.

Equilibrium swelling was determined using fully cured and thoroughly washed specimens. The hydrogels were first dried to constant mass and then immersed in the reference synthetic brine containing 60,000 mg/L total dissolved solids at 25 °C until the specimen mass varied by less than 0.5% over 6 h. The swollen specimens were gently blotted to remove surface liquid and immediately weighed. The equilibrium swelling ratio (*Q*_e_) and equilibrium water content (EWC) were calculated from the dry and equilibrium-swollen masses according to the equations given in Section 4.11. Three independently prepared specimens were tested for each formulation, and the results are reported as mean ± standard deviation.

### 4.5. Spectroscopic and Initial Multiscale Structural Characterization

The chemical environment and inorganic-phase composition of CN, Si-NC, and Lap-DN were characterized by Fourier-transform infrared spectroscopy (FTIR) and X-ray photoelectron spectroscopy (XPS). Fully cured hydrogels were washed, rapidly frozen, and lyophilized before analysis. FTIR spectra were collected over 4000–500 cm^−1^ at a spectral resolution of 4 cm^−1^. The spectra were baseline corrected and normalized before comparison. The amide-I region (1725–1605 cm^−1^) was additionally examined, and the positions of the principal carbonyl-related bands were reported as approximate band maxima, with interpretation constrained by the spectral resolution of 4 cm^−1^. Vertical offsets applied to the spectra in Figure 4 were used solely for visualization.

XPS survey spectra were acquired over a binding-energy range sufficient to quantify the major surface elements, and the binding-energy scale was calibrated using the C 1s C–C/C–H component at 284.8 eV. Elemental atomic concentrations were calculated from the integrated peak areas using the corresponding instrumental sensitivity factors. Si and Mg contents were used to distinguish the nano-SiO_2_- and Laponite-containing systems. Elemental concentrations below the instrumental detection limit were reported as ND rather than zero.

The initial dispersion state of Laponite was examined by transmission electron microscopy (TEM). A dilute aliquot of the polymer-containing Laponite dispersion was collected immediately before initiation, deposited onto carbon-coated copper grids, and dried under ambient conditions prior to imaging. Several randomly selected regions were examined to identify isolated platelets, short stacks, and residual tactoid-like domains.

For X-ray diffraction (XRD), Laponite XLG powder and identically prepared lyophilized hydrogel specimens were analyzed using Cu Kα radiation (λ = 0.15406 nm). Low-angle diffraction patterns were collected over a 2*θ* range of approximately 3–30° with a step size of 0.02°. The position of the residual Laponite basal reflection was used to calculate an apparent dried-state *d*_001_ spacing according to Bragg’s law, while peak area and full width at half maximum (FWHM) were obtained after consistent baseline correction. Because hydrogel specimens required freezing and lyophilization before XRD measurement, the calculated *d*_001_ values and associated peak parameters were used only for comparative analysis of residual layer ordering among identically prepared specimens. They were not interpreted as direct measurements of the native hydrated interlayer spacing or as unique evidence of in situ platelet rearrangement. The same peak-analysis procedure was subsequently applied to thermally treated Lap-DN samples in Section 4.8.

For cryo-SEM observation, fully hydrated CN, Si-NC, and Lap-DN specimens were rapidly cryo-fixed to minimize redistribution of the aqueous phase, fractured under cryogenic conditions, and sputter-coated with a thin conductive layer before imaging. Pore-network analysis was performed on micrographs acquired at identical magnification. Individual void regions were segmented using the same image-processing threshold, and the equivalent pore diameter,

(8) $$d_{\mathrm{eq}}\;=\;2\sqrt{\frac{A}{\pi }}$$

was calculated from the projected pore area *A*. At least five randomly selected fields were analyzed for each independently prepared hydrogel batch. Mean pore diameter and the pore-size coefficient of variation were calculated first for each batch, and the batch-level values from three independent preparations were used for statistical comparison to avoid treating individual pores as independent experimental replicates.

### 4.6. Cyclic Thermal Exposure and Artifact-Control Protocols

Cyclic hydrothermal treatments were performed on fully cured and brine-equilibrated CN, Si-NC, and Lap-DN specimens in the reference synthetic brine containing 60,000 mg/L total dissolved solids using the sealed high-pressure reactor described in Section 4.1.2. The specimens remained in the aqueous brine phase throughout the prescribed hydrothermal treatment. No external gas pressure was applied; instead, the reactor was operated under autogenous hydrothermal pressure generated during heating of the closed aqueous system. Pressure was therefore not independently imposed or used as an experimental variable. For thermodynamic reference, the saturation pressure of pure water at 180 °C is approximately 1.003 MPa absolute; however, this value is not assigned as the actual reactor pressure because the present experiments employed saline solution and the autogenous pressure also depends on the gas–liquid volume distribution within the sealed reactor. For each cycle, the specimens were heated from 25 °C to 180 °C, and the 30 min high-temperature holding period was initiated only after the internal temperature reached 180 ± 2 °C. After the holding period, the system was cooled to 25 ± 2 °C, after which a 30 min recovery period was applied. Heating and cooling transition times were recorded separately and were not included in either the high-temperature holding period or the recovery period. Unless otherwise specified, five cycles were performed, corresponding to a cumulative isothermal holding time of 150 min at 180 °C.

To distinguish cycle-induced reinforcement from dehydration, continued post-curing, and conventional thermal aging, four thermal-history groups were established. Initial specimens received no additional thermal treatment after curing and equilibration. Time-matched specimens were maintained at 25 °C for the same total elapsed time as that required to complete the five-cycle treatment, including the measured heating and cooling transition periods. Isothermal-180 specimens were heated to 180 °C using the same initial heating procedure and continuously maintained at 180 ± 2 °C for 150 min. After cooling, they were kept at 25 °C until their total elapsed time matched that of the cyclic group. Cyclic-180 specimens underwent five consecutive heating–holding–cooling–recovery sequences as described above. Separate specimens from three independently prepared batches were used for each treatment condition.

Parallel specimens from the same independently prepared batch were used for the as-recovered and re-equilibrated water-content measurements. One set was analyzed immediately after the assigned thermal treatment, whereas the second set was re-equilibrated in fresh reference brine at 25 °C for 24 h before measurement. The wet-basis water content was calculated from the corresponding wet and dry specimen masses as described in Section 4.11. Rheological comparisons among different thermal histories were performed only after this standardized re-equilibration step to minimize differences arising from transient dehydration. This procedure was designed to compare the persistence of thermal-history-dependent mechanical changes at a common hydration state rather than to preserve the instantaneous high-temperature network configuration. Because fresh-brine equilibration may itself allow additional swelling, polymer-chain relaxation, and reorganization of reversible physical constraints, the resulting rheological state is referred to throughout the manuscript as the post-treatment, re-equilibrated state. All comparison groups were subjected to the identical 24 h re-equilibration protocol so that any such recovery-related restructuring occurred under the same external hydration condition.

Gel fraction and extractable residual monomer content were determined using separate specimens from the same treatment groups. For gel-fraction measurements, the dried hydrogel was extracted in deionized water for 72 h with periodic renewal of the extraction medium and subsequently dried to constant mass. For Si-NC and Lap-DN, the known inorganic nanophase contribution was subtracted from the residual dry mass before calculating the polymer gel fraction. Residual AM, NVP, and NaAMPS were extracted from the treated hydrogels using deionized water at a fixed sample-to-extractant ratio and quantified against external calibration standards. The total extractable residual-monomer content was calculated from the summed masses of the three quantified monomers and expressed as a percentage of the initial total monomer mass used to prepare the corresponding specimen.

For cycle-dependent rheological measurements, specimens were subjected to 0–5 thermal cycles under the same protocol. After the prescribed number of cycles, the samples were re-equilibrated to the reference hydration state and tested at 25 °C, 1% strain, and 1 rad·s^−1^. Frequency sweeps of Lap-DN before thermal cycling and after the fifth cycle were performed over 0.1–100 rad·s^−1^ at 1% strain. The storage-modulus reinforcement ratio was calculated from the storage modulus after thermal treatment relative to the corresponding initial value, as defined in Section 4.11.

Dynamic recovery after cyclic thermal treatment was evaluated by alternating low- and high-strain oscillatory deformation at 25 °C and 1 rad·s^−1^. After equilibration at 1% strain, the strain amplitude was switched to 300% for 60 s to disrupt the physical network and then returned to 1% for 180 s to allow structural recovery. Five consecutive damage–recovery cycles were applied. The modulus-recovery ratio (*R*_G_) for each cycle was determined from the recovered *G*′ during the low-strain interval relative to the pre-damage *G*′, with the calculation given in Section 4.11. Three independently prepared specimens were tested for each hydrogel formulation.

### 4.7. Compression, Energy-Dissipation, and Mechanical-Recovery Tests

Unconfined compression tests were performed on cylindrical hydrogel specimens with a diameter of 15 mm and an initial height of 10 mm. Before testing, CN, Si-NC, and Lap-DN specimens were subjected to the prescribed number of thermal cycles according to Section 4.6 and subsequently re-equilibrated to the reference hydration state. The specimens were placed between parallel compression platens, with a thin layer of reference brine applied to the contact surfaces to minimize interfacial friction. Compression was conducted at 25 °C using a constant crosshead speed of 2 mm/min.

For monotonic compression, specimens were compressed to 70% engineering strain. The compressive stress at 50% strain, *σ*_50_, was used as a representative finite-deformation strength parameter. The apparent compressive modulus, *E*_c_, was obtained from the linear regression of the stress–strain curve over the 5–15% strain interval. Representative stress–strain curves were obtained for Lap-DN after 0, 1, 3, and 5 thermal cycles, whereas *σ*_50_ and *E*_c_ were determined for CN, Si-NC, and Lap-DN after 0–5 cycles using independently prepared specimens.

Loading–unloading tests were performed to quantify mechanical energy dissipation. Specimens were compressed from 0 to 60% strain and immediately unloaded to zero force at the same crosshead speed. The dissipated energy density, *U*_d_, was calculated from the area enclosed by the loading–unloading hysteresis loop, while the energy-dissipation ratio, *η*_d_, was defined as the ratio of *U*_d_ to the total mechanical work during loading. The corresponding equations are given in Section 4.11.

Mechanical recovery after large deformation was evaluated using specimens that had completed five thermal cycles. Each specimen was compressed once to 60% strain and unloaded to zero force, after which it was allowed to recover in the reference synthetic brine at 25 °C. Residual compressive strain was recorded after recovery periods of 0, 0.5, 2, 6, 12, and 24 h. For strength-recovery measurements, parallel specimens subjected to the same 60% damaging compression were allowed to recover for the designated period and were then recompressed under the same conditions. The compressive stress at 50% strain during reloading, *σ*_50,rec_, was compared with the corresponding pre-damage value, *σ*_50,pre_, to calculate the compressive-strength recovery ratio *R*_σ_. Three independently prepared specimens were tested for each formulation and recovery time.

### 4.8. Structural Evolution, Water Retention, Volume Stability, and Salinity Tests

Structural evolution of Lap-DN was evaluated after different thermal histories using the cyclic-treatment protocol described in Section 4.6. Five states were examined: the initial hydrogel, continuous exposure at 180 °C for 150 min (Isothermal-180), and hydrogels recovered after 1, 3, and 5 thermal cycles. After treatment, specimens used for structural comparison were re-equilibrated in the reference synthetic brine at 25 °C to minimize differences caused by transient dehydration. For XRD analysis, excess surface liquid was removed before rapid freezing and lyophilization. Low-angle diffraction patterns were collected and analyzed using the same conditions and baseline-correction procedure described in Section 4.5. The *d*_001_ spacing, integrated area, and full width at half maximum (FWHM) of the residual Laponite basal reflection were determined for each thermal state. Peak area and FWHM were normalized to the corresponding values of the initial Lap-DN specimen.

Changes in the hydrated pore architecture were characterized by cryo-SEM. Re-equilibrated specimens were rapidly cryo-fixed and prepared using the same procedure as described in Section 4.5. Images were acquired at identical magnification and analyzed using a common segmentation protocol. Mean equivalent pore diameter and pore-size coefficient of variation were determined for the initial, Isothermal-180, Cycle-1, Cycle-3, and Cycle-5 states. At least five randomly selected fields were analyzed for each independently prepared batch, and batch-level averages from three independent preparations were used for statistical comparison.

Water retention and dimensional stability during thermal cycling were evaluated for CN, Si-NC, and Lap-DN. Fully equilibrated cylindrical specimens were subjected to 0–5 thermal cycles at 180 °C in the reference synthetic brine. Separate specimens were terminated after each prescribed number of cycles to avoid interference from destructive drying measurements. After each endpoint, surface liquid was removed gently and the wet mass and specimen dimensions were recorded immediately. The specimens were subsequently dried to constant mass for determination of the retained water mass. Water-retention ratio (*W*_r_) was calculated relative to the initial water content of the corresponding specimen, whereas volume-retention ratio (*V*_r_) was calculated from the specimen volume after thermal treatment relative to its initial volume. The corresponding equations are given in Section 4.11.

Salinity tolerance was investigated using synthetic brines with nominal total dissolved-solids concentrations of 0, 30,000, 60,000, 100,000, and 150,000 mg/L. The 60,000 mg/L brine corresponded to the reference composition listed in Table 2. The other saline solutions were prepared by proportionally scaling all constituent salts while maintaining the same relative salt composition, whereas deionized water was used for the 0 mg/L condition. Hydrogel specimens were pre-equilibrated in the corresponding solution before thermal treatment and then subjected to five heating–cooling cycles using the same protocol as described in Section 4.6.

After the fifth cycle, specimens used for rheological measurements were re-equilibrated in fresh solution of the same salinity and tested at 25 °C, 1% strain, and 1 rad·s^−1^. The cycle-5 modulus-retention ratio was calculated as G_5_′/G_0_′, where both values were obtained at the same salinity. For volume-stability measurements, the specimen volume immediately after the fifth cycle was compared with its corresponding initial volume at the same salinity. Three independently prepared specimens were analyzed for each hydrogel formulation, thermal state, and salinity condition.

### 4.9. Fracture-Sealing Tests Under Steam Exposure

Fracture-sealing performance was evaluated using a high-temperature variable-aperture fracture-flow cell. The effective fracture-flow section had a constant length L and width *W*, while the nominal aperture was adjusted using precision spacers to 0.30, 0.50, 0.80, 1.20, or 1.60 mm. The resulting aperture therefore represents a nominal geometrically controlled value intended to isolate the influence of fracture opening. The present protocol did not independently vary fracture-surface roughness, spatial aperture heterogeneity, mineral-surface composition, or prior crude-oil conditioning; these factors were outside the scope of the controlled comparative tests. Accordingly, the fracture model was used to compare the intrinsic sealing responses of CN, Si-NC, and Lap-DN under identical geometric and fluid conditions rather than to reproduce the full structural and interfacial complexity of a natural reservoir fracture. Before each experiment, the fracture cell was cleaned, assembled at the prescribed aperture, vacuumed, and saturated with the reference synthetic brine containing 60,000 mg/L total dissolved solids. The initial fracture conductivity was measured under a low differential-pressure condition using the reference brine before hydrogel placement.

Fresh CN, Si-NC, or Lap-DN precursor prepared according to Section 4.2 was injected into the fracture cell before the onset of macroscopic gelation. Approximately 1.5 fracture volumes of precursor were introduced to ensure complete displacement of the resident brine and uniform occupation of the fracture space. The inlet and outlet valves were then closed, and the precursor was polymerized in situ at 70 °C for 12 h. After gel formation, the fracture cell was flushed slowly with the reference brine under a differential pressure well below the expected breakthrough pressure to remove unconfined material. The confined hydrogel was then maintained in the reference brine at 25 °C for 24 h before steam exposure to establish a standardized initial hydration condition.

The gel-filled fracture was subsequently exposed to saturated steam at 180 °C for 30 min. For saturated water vapor, 180 °C corresponds to an equilibrium saturation pressure of approximately 1.003 MPa absolute; this steam-exposure stage was distinct from the subsequent pressurized liquid-flow evaluation. After steam exposure, the flow path was switched to the reference synthetic brine while the fracture cell was maintained at 180 °C. A downstream back-pressure regulator was set to 2.0 MPa to suppress flashing and maintain single-phase liquid flow during the hydraulic evaluation. Accordingly, the 2.0 MPa back pressure refers only to the post-steam brine-flow and breakthrough measurements and should not be interpreted as the pressure of the preceding saturated-steam exposure. After the temperature and downstream pressure had stabilized at 180 ± 2 °C and 2.0 ± 0.05 MPa, respectively, the reference brine was injected at a constant flow rate of 2.0 mL/min. Upstream and downstream pressures were recorded continuously, and the differential pressure was calculated as Δ*P* = *P*_up_−*P*_down_. Breakthrough was defined as the first sustained downstream flow response accompanied by an irreversible decrease in Δ*P*. The maximum differential pressure immediately preceding this event was recorded as the breakthrough pressure, *P*_b_. Representative pressure–time histories were obtained for a fracture aperture of 0.80 mm.

Fracture conductivity was measured both before hydrogel placement and after establishment of the hydrogel seal under a non-destructive, low-differential-pressure single-phase brine-flow condition. The effective flow section of the fracture cell had a constant length of *L* = 100 mm and width of *W* = 25 mm, while the nominal aperture was independently adjusted to 0.30, 0.50, 0.80, 1.20, or 1.60 mm using precision spacers. The same effective length, width, and prescribed aperture were maintained for the paired pre-placement and post-sealing measurements.

For the conductivity measurement, the fracture cell was maintained at 180 ± 2 °C, and the downstream pressure was controlled at *P*_down_ = 2.00 ± 0.05 MPa using the back-pressure regulator to maintain the reference brine in the single-phase liquid state. A constant reference-brine flow rate of *Q* = 0.50 mL/min was applied, which was one quarter of the 2.0 mL/min injection rate used in the subsequent breakthrough-pressure test. The conductivity measurement was restricted to a stabilized differential pressure not exceeding 0.10 MPa to minimize hydraulic disturbance of the established hydrogel seal. The upstream and downstream pressures were recorded continuously, and a stable flow state was considered to have been reached when the variation in differential pressure remained within 2% for at least 5 min.

The stabilized differential pressure used for conductivity calculation was defined as Δ*P* = *P*_up_ − *P*_down_, where *P*_up_ and *P*_down_ are the stabilized upstream and downstream pressures, respectively. No abrupt pressure decay or other indication of seal destabilization was allowed during this low-flow measurement. The conductivity test was therefore performed independently from the subsequent breakthrough-pressure test and was not used to determine *P*_b_.

The initial fracture conductivity, *C*_f,0_, was determined in the clean brine-saturated fracture before hydrogel placement. Following precursor injection, in situ gelation, and steam exposure, the post-sealing conductivity, *C*_f_, was determined using the same effective fracture dimensions, prescribed aperture, temperature, reference-brine composition, downstream-pressure condition, and low-flow conductivity-test protocol. Thus, *C*_f,0_ and *C*_f_ constituted paired measurements for the same fracture geometry. Fracture conductivity was calculated from the stabilized *Q*–Δ*P* response according to Equation (17), and the corresponding fracture-conductivity reduction, *R*_C_, was subsequently calculated according to Equation (18). Three independently prepared sealing specimens were tested for each hydrogel formulation and fracture aperture.

### 4.10. Cyclic Steam Impact–Recovery–Resealing Tests

Cyclic resealing experiments were performed using the fracture-flow system described in Section 4.9 with the fracture aperture fixed at 1.20 mm. CN, Si-NC, and Lap-DN precursors were placed and polymerized in situ using the same procedure as in the single-exposure sealing tests. Each independently prepared sealing body remained in the same fracture throughout the entire cyclic experiment; no additional precursor or hydrogel was introduced, and the fracture cell was not opened between cycles.

Each cycle consisted of a high-temperature steam-impact stage at 180 °C under the same steam condition described in Section 4.9, followed by depressurization and recovery at 25 °C for 30 min. After thermal equilibration at 180 °C, the flow path was switched to the reference synthetic brine (60,000 mg/L). The downstream pressure was maintained at 2.0 ± 0.05 MPa using the back-pressure regulator during the subsequent liquid-phase hydraulic challenge, and the sealing body was challenged at a constant injection rate of 2.0 mL/min. Differential pressure was recorded continuously until sustained downstream breakthrough occurred. The maximum differential pressure immediately preceding breakthrough was taken as *P*_b_. After breakthrough, the system was depressurized and cooled to 25 °C for 30 min without disturbing the sealing body, after which the cell was reheated and the next challenge was conducted. Five consecutive breakthrough–recovery–resealing cycles were completed for each specimen.

Fracture conductivity was evaluated under the same non-destructive, low-differential-pressure single-phase brine condition defined in Section 4.9. For each cycle, conductivity was measured after the prescribed recovery/resealing step and before the subsequent high-pressure breakthrough challenge. The fracture dimensions remained unchanged throughout the five-cycle experiment, and *C*_f_ for each resealing state was calculated from the stabilized *Q*–Δ*P* response using Equation (17). The corresponding fracture-conductivity reduction, *R*_C_, was calculated relative to the initial unsealed fracture conductivity according to Section 4.11. The first-cycle values were obtained under the same fracture aperture, temperature, fluid composition, and hydraulic conditions used in the single-exposure experiments of Section 4.9.

Recovery kinetics after breakthrough were examined separately using parallel sealing specimens. Each specimen was first subjected to the same cyclic protocol through a standardized fifth-cycle breakthrough and was then allowed to recover at 25 °C for 0, 10, 30, 60, or 120 min. After the designated recovery interval, the fracture cell was reheated to 180 °C and challenged again under the same hydraulic conditions. The breakthrough-pressure recovery ratio, *R*_P_, was calculated from the recovered breakthrough pressure relative to the corresponding pre-recovery breakthrough pressure, as defined in Section 4.11. Independent specimens were used for each recovery time to prevent repeated re-breaking from influencing the recovery-time series. Three independently prepared sealing specimens were tested for each formulation and condition.

### 4.11. Calculations and Statistical Analysis

The equilibrium swelling ratio (*Q*_e_) and equilibrium water content (*EWC*) were calculated from the equilibrium-swollen mass (*m*_e_) and dry mass (*m*_d_) as

(9) $$Q_{e}\;=\;\frac{m_{e}\;-\;m_{d}}{m_{d}},\;EWC\;=\;\frac{m_{e}\;-\;m_{d}}{m_{e}}\;\times \;100\%$$

where *Q*_e_ is expressed in g/g.

For nanoparticle-containing hydrogels, the polymer gel fraction was corrected for the inorganic nanophase according to

(10) $$GF_{\mathrm{polymer}}\;=\;\frac{m_{d,e}\;-\;m_{\mathrm{inorg}}}{m_{d,0}\;-\;m_{\mathrm{inorg}}}\;\times \;100\%$$

where *m*_d,0_ and *m*_d,e_ are the dry masses before and after aqueous extraction, respectively, and *m*_inorg_ is the mass of Laponite or nano-SiO_2_ contained in the specimen. For CN, *m*_inorg_ = 0. Wet-basis water content in the artifact-control experiments was calculated from the water mass relative to the total wet specimen mass. The extractable residual-monomer content was expressed as the summed mass of residual AM, NVP, and NaAMPS relative to the initial total monomer charge of the corresponding specimen.

The storage-modulus self-reinforcement index after the nth thermal cycle was defined as

(11) $$SRI_{G}(n)\;=\;\frac{G_{n}^{\prime }}{G_{0}^{\prime }}$$

where *G*_0_′ and *G*_n_′ are the storage moduli before thermal treatment and after the nth cycle, respectively, measured under the same hydration and rheological conditions. For the salinity-dependent experiments, 100 × *SRI*_G_(5) was reported as the cycle-5 modulus-retention percentage, with *G*_0_′ and *G*_5_′ always measured at the same salinity.

The modulus-recovery ratio obtained from the step-strain experiments was calculated as

(12) $$R_{G}\;=\;\frac{G_{\mathrm{rec}}^{\prime }}{G_{\mathrm{pre}}^{\prime }}\;\times \;100\%$$

where *G*_pre_′ is the modulus immediately before high-strain disruption and *G*_rec_′ is the recovered modulus after returning to the low-strain condition.

Mechanical self-reinforcement was quantified using the compressive stress at 50% strain:

(13) $$SRI_{\sigma }(n)\;=\;\frac{\sigma_{50,n}}{\sigma_{50,0}}$$

where *σ*_50,0_ and *σ*_50,n_ represent the corresponding stresses before and after the nth thermal cycle. Engineering stress and strain were calculated using the initial specimen dimensions, and the apparent compressive modulus *E*_c_ was obtained from the slope of the stress–strain curve between 5% and 15% strain.

The dissipated energy density and energy-dissipation ratio were obtained from the loading–unloading hysteresis loop:

(14) $$U_{d}\;=\;\int_{0}^{\varepsilon_{\max}}\sigma_{\mathrm{load}}d\varepsilon 3\;-\;\int_{0}^{\varepsilon_{\max}}\sigma_{\mathrm{unload}}d\varepsilon ,\;\eta_{d}\;=\;\frac{U_{d}}{U_{\mathrm{load}}}\;\times \;100\%$$

where *U*_load_ is the area under the loading curve up to the prescribed maximum strain.

The compressive-strength recovery ratio after a recovery period t was defined as

(15) $$R_{\sigma }(t)\;=\;\frac{\sigma_{50,\mathrm{rec}}(t)}{\sigma_{50,\mathrm{pre}}}\;\times \;100\%$$

where *σ*_50,pre_ and *σ*_50,rec_(*t*) are the compressive stresses at 50% strain before the damaging compression and after recovery for the specified period *t*, respectively.

Water-retention and volume-retention ratios during thermal cycling were calculated as

(16) $$W_{r}(n)=\frac{m_{w,n}}{m_{w,0}}\times 100\%,\;V_{r}(n)=\frac{V_{n}}{V_{0}}\times 100\%$$

where *m*_w_ = *m*_wet_ − *m*_dry_, and V_0_ and V_n_ are the specimen volumes before and after the nth thermal cycle, respectively.

For the single-phase fracture-flow measurements, fracture conductivity was calculated as

(17) $$C_{f}\;=\;\frac{Q\mu L}{W\Delta P}$$

where *C*_f_ is the fracture conductivity, defined here as the permeability–aperture product (*k*_f_*b*); *k*_f_ is the effective fracture permeability; *b* is the fracture aperture; *Q* is the stabilized volumetric brine flow rate; *μ* is the dynamic viscosity of the reference brine at the measurement temperature; *L* and *W* are the effective fracture-flow length and width, respectively; and Δ*P* = *P*_up_ − *P*_down_ is the stabilized differential pressure across the fracture during the non-destructive low-pressure conductivity measurement. Equation (17) follows the one-dimensional Darcy form *Q* = *C*_f_*W*Δ*P*/(*μL*). Because *C*_f_ is defined as *k*_f_*b*, the fracture aperture is incorporated into the conductivity term and therefore does not appear as a separate multiplier in Equation (17). All quantities were converted to SI units before calculation.

Fracture-conductivity reduction was calculated as

(18) $$R_{C}\;=\;\left(1\;-\;\frac{C_{f}}{C_{f,0}}\right)\;\times \;100\%$$

where *C*_f,0_ and *C*_f_ are the fracture conductivities measured for the same prescribed fracture aperture before hydrogel placement and after establishment of the steam-exposed hydrogel seal, respectively. Both measurements were performed using the same effective fracture dimensions, reference-brine composition, temperature, downstream-pressure condition, and low-differential-pressure conductivity-test procedure. Therefore, *R*_C_ quantifies the reduction of hydraulic conductivity caused by the established hydrogel seal and is independent of the breakthrough-pressure metric *P*_b_.

For cyclic resealing experiments, breakthrough-pressure retention and post-breakthrough pressure recovery were calculated as

(19) $$R_{P,n}\;=\;\frac{P_{b,n}}{P_{b,1}}\;\times \;100\%,\;R_{P}(t)\;=\;\frac{P_{b,\mathrm{rec}}(t)}{P_{b,\mathrm{pre}}}\;\times \;100\%$$

where *P*_b,n_ is the breakthrough pressure during the nth resealing cycle, *P*_b,1_ is the first-cycle value, *P*_b,pre_ is the breakthrough pressure before the standardized recovery period, and *P*_b,rec_(*t*) is the breakthrough pressure measured after recovery for time *t*.

The Laponite basal spacing was calculated from the XRD peak position using Bragg’s law, and the basal-peak area and FWHM were normalized to those of the initial Lap-DN specimen. Equivalent pore diameter was calculated from segmented pore area as described in Section 4.5; individual pores and microscopic fields were treated as subsamples rather than independent replicates.

Unless otherwise specified, quantitative results are presented as mean ± standard deviation from three independently prepared hydrogel batches or sealing specimens (*n* = 3). Individual pores and microscopic fields obtained from the same hydrogel batch were treated as subsamples rather than independent replicates. For independent multi-group comparisons, one-way analysis of variance followed by Tukey’s multiple-comparison test was applied, with *p* < 0.05 considered statistically significant. For the cyclic resealing data in Figure 13b,c, each sealing specimen was followed repeatedly over cycles 1–5; these cycle-resolved measurements were therefore treated as repeated observations from the same specimen and are presented descriptively as mean ± standard deviation without applying one-way ANOVA across cycle number. The recovery-time data in Figure 13d were obtained from independent parallel specimens at each recovery interval and were analyzed as independent groups where statistical comparison was required. The structure–property and property–sealing trajectories in Figure 14 were interpreted descriptively and were not subjected to inferential linear-regression analysis.

## Figures and Tables

**Table 1:** Contextual comparison of Lap-DN with representative high-temperature reservoir gel systems reported in the literature.

System	Material/Configuration	Reported Thermal and Saline Conditions	Representative Reported Performance	Principal Characteristic
HT-PPG [10]	Ultra-high-temperature preformed particle gel	150 °C; including 2 wt% KCl brine	*G*′ > 3 kPa at approximately 90% water content; >30-fold swelling; hydrothermal stability for >18 months in 2 wt% KCl at 150 °C; successful fractured-core plugging	Long-term hydrothermal stability
Alg-IPNG [11]	Self-healing P(AM-co-VAc)/alginate interpenetrating particle gel	Up to 110 °C	*G*′ up to 1.89 kPa; self-healing initiation within approximately 38–60 h; thermal stability for 180 days	Ion-mediated self-healing and reinforcement
Laponite hydrogel [18]	Chemically crosslinked acrylamide/Laponite hydrogel	Rheological evaluation at 50 °C; temperature and salinity effects investigated	Improved thixotropy, network uniformity, and rapid structural recovery after shear; increasing temperature and salt concentration weakened the Laponite contribution	Laponite-mediated thixotropic recovery
Cs/PAMBA PPG [19]	Chitosan-grafted polyacrylamide PPG	≤130 °C; salinity tolerance reported up to 200,000 ppm	*G*′ of approximately 3.70–22.91 kPa in saline solution with TDS of approximately 67.3 g/L; good long-term high-temperature stability	High salinity tolerance
EF-HT-RPPG [23]	Lysine-crosslinked re-crosslinkable PPG	Re-crosslinking at 80–130 °C; >350 days stability at 130 °C	*G*′ ≈ 0.477 kPa at a swelling ratio of 10; fracture breakthrough-pressure gradient ≈1.58 MPa/m; effective fracture injectivity and plugging	High-temperature re-crosslinking
Lap-DN, this work	Covalent–physical Laponite nanocomposite hydrogel	Five cyclic exposures at 180 °C; 60,000 mg/L reference brine; mechanical retention evaluated up to 150,000 mg/L	*G*′ increased from 74.4 to 89.1 kPa; *P*_b_ increased from 4.29 to 4.55 MPa over five cycles in a 1.20 mm fracture; *R*_C_ remained 93.5%; 95.0% of pressure resistance recovered within 30 min	Cycle-triggered self-reinforcement and rapid repeated resealing

Note: Direct numerical ranking among the reported systems should be avoided because the gel geometry, swelling state, brine composition, thermal history, and mechanical or fracture-testing protocols differ among studies. The comparison is intended to identify differences in functional emphasis and operating conditions rather than to establish universal superiority of one material.

**Table 2:** Composition of the reference synthetic brine.

Salt	Concentration/(mg/L)	Mass Fraction of Total Salts/%
NaCl	48,000	80.0
KCl	1200	2.0
CaCl_2_	4800	8.0
MgCl_2_·6HO	4200	7.0
Na_2_SO_4_	1800	3.0
Total	60,000	100.0

**Table 3:** Formulation of the principal hydrogel precursor systems. Basis: 100 g final precursor.

Component	Formulation Basis	CN/g	Si-NC/g	Lap-DN/g
NaAMPS	40 mol% of total monomers	12.88	12.88	12.88
NVP	20 mol% of total monomers	3.12	3.12	3.12
AM	40 mol% of total monomers	4.00	4.00	4.00
Total monomers	20.0 wt% of final precursor	20.00	20.00	20.00
MBAA	0.20 wt% of total monomer mass	0.040	0.040	0.040
APS	0.50 wt% of total monomer mass	0.100	0.100	0.100
Hydrophilic nano-SiO_2_	1.00 wt% of final precursor	—	1.00	—
Laponite XLG	1.00 wt% of final precursor	—	—	1.00
Deionized water ^a^	Added to a final precursor mass of 100 g	79.86	78.86	78.86
Total precursor	—	100.00	100.00	100.00

^a^ The water used to prepare the MBAA and APS stock solutions was included in the total deionized-water mass. Note: The NaAMPS/NVP/AM ratio is expressed on a molar basis. MBAA and APS contents are expressed relative to the total monomer mass, whereas nano-SiO_2_ and Laponite XLG contents are expressed relative to the final precursor mass.

## Data Availability

The data presented in this study are available on request from the corresponding author.
